# Epigenetic and Neural Circuitry Landscape of Psychotherapeutic Interventions

**DOI:** 10.1155/2017/5491812

**Published:** 2017-05-25

**Authors:** Christopher W. T. Miller

**Affiliations:** University of Maryland School of Medicine, 701 W. Pratt St., 4th Floor, Baltimore, MD 21201, USA

## Abstract

The science behind psychotherapy has garnered considerable interest, as objective measures are being developed to map the patient's subjective change over the course of treatment. Prenatal and early life influences have a lasting impact on how genes are expressed and the manner in which neural circuits are consolidated. Transgenerationally transmitted epigenetic markers as well as templates of enhanced thought flexibility versus evasion can be passed down from parent to child. This influences gene expression/repression (impacting neuroplasticity) and kindling of neurocircuitry which can perpetuate maladaptive cognitive processing seen in a number of psychiatric conditions. Importantly, genetic factors and the compounding effects of early life adversity do not inexorably lead to certain fated outcomes. The concepts of vulnerability and resilience are becoming more integrated into the framework of “differential susceptibility,” speaking to how corrective environmental factors may promote epigenetic change and reconfigure neural templates, allowing for symptomatic improvement. Psychotherapy is one such factor, and this review will focus on our current knowledge of its epigenetic and neurocircuitry impact.

## 1. Introduction

The effects of psychotherapy from a clinical perspective are growing in evidence base, particularly given the desire within the scientific community to translate concepts which many times are subjective and abstract into demonstrable and replicable effects. This has gained substantial ground recently, as the benefits of psychotherapy have been demonstrated on multiple levels, including its epigenetic, neurocircuitry, and neuroendocrine influences. Also, animal models have shown the benefits of continued environmental enrichment (EE) on psychopathological phenotypes, which carries exciting translational value, particularly given that some of these studies utilize animals which are genetically bred to exhibit maladaptive phenotypes. Thus, the environment can continue to influence behavioral paradigms throughout the life cycle. The premise of most forms of psychotherapy highlights the importance of the therapeutic alliance and the development of a rapport that feels containing for the patient. Indeed, the “Dodo Bird Verdict,” positing that in the end all forms of psychotherapy have some degree of empirical equivalence [1, 2], has garnered considerable controversy [3], but on a neuroscientific level there may be some value to it, given that much of what has been shown as the effects of continued psychotherapeutic action has been activation of more interpersonally attuned areas of the brain, as well as greater expression of genes allowing for plasticity in areas involved with higher cognitive processing and with fine-tuning the activity of subcortical areas. Neuroendocrine and neurocircuitry templates established early in life will be the default manner of negotiating environmental stimuli, and conditioned responses, especially as they pertain to interpersonal models of interacting, will arise within the therapeutic dyad (i.e., the therapist and patient). Assessing the patient's symptomatology and personality structure in light of early life experience has been enriched by research investigating how their individual make-up came to be, and reshaping of endocrine responses and select activation of certain neural networks, in particular when faced with life stressors, is a powerful indicator of therapeutic response. This paper will aim to outline how early life experience lays down this template, which in many ways is enduring and very difficult to undo, and how the environment factors can foster significant change throughout one's life.

## 2. Prenatal and Early Life Influence

Most central nervous system (CNS) neurogenesis occurs over a period spanning just over two years, and most telencephalic neurons are generated before birth [4]. Contingent and appropriate responses from the infant's primary caregiver are crucial in facilitating CNS development, and the more interpersonally attuned right hemisphere shows a more accelerated growth during the late fetal and early postnatal periods [5, 6]. Attunement to the child also informs how he or she will internalize the model for social interactions; early life neglect and abuse can lead to a plethora of psychiatric and somatic conditions, including growth delays, immune dysregulation, low levels of oxytocin, and impairment in social reciprocity [7–10]. Thus, the “psychobiologically attuned caregiver” has a pivotal role in regulating an infant's brain development [11]. In ideal situations, the caregiver will be attuned to the child and able to provide comfort during distressing states, something which will allow for the child to emerge with a basic sense of safety and trust, in particular as motor skills are furthered and exploration of the world is the next task to face, something which may be exciting or terrifying.

### 2.1. Genetic Considerations

#### 2.1.1. Vulnerability Genes

Genetic vulnerability is a topic of some controversy, particularly as we learn more about the complex interaction between an individual's genetic endowment and how the environment influences expression. A number of genes which have been termed “vulnerability genes” have been identified and replicated in human studies as conferring a particular risk of psychopathology. The counterpoint to this will be presented later in the paper, underlining concept of “differential susceptibility” [12, 13], a hypothesis which posits that there is varying susceptibility of individuals to the effects of the environment depending on genetic make-up. As such, some individuals would display worse outcomes in negative environments but flourish more in positive environments, highlighting the “plastic” nature of their responses [14]. The genes that will be discussed in this paper are primarily those upon which psychotherapy has been shown to have an impact. Caspi et al. outlined two particular genes in groundbreaking studies which highlighted one's susceptibility to behavioral and emotional dysregulation, when coupled with an adverse early environment [15, 16]. The first of the studies was performed in human subjects of both genders (with a sample of roughly 1,000 individuals), following them across multiple time points throughout their lives, and demonstrated that a low-functioning allele of monoamine oxidase A (MAO-A) was associated with numerous adverse outcomes later in life, relating particularly to conduct disorder, antisocial personality disorder, violence, and incarceration [15]. As MAO-A is responsible for the breakdown of serotonin, norepinephrine, and dopamine, suboptimal functioning can lead to an excess in neurotransmitter availability. An excess of serotonin can be associated with amygdala hyperreactivity and altered threat processing [17]. Also, elevated levels of norepinephrine and dopamine have been associated with greater externalizing behaviors and aggressiveness [18], more pronounced when immersed in a threatening and unpredictable environment. Importantly, MAO-A is an X-linked gene, and males carrying a hypoactive allele may have in effect a knockout gene.

Another vulnerability gene is the Solute Carrier Family 6, member 4 (SLC6A4), which codes for the serotonin transporter (5-HTT or SERT). The short (s) allele of the serotonin transporter-linked polymorphic region (5-HTTLPR) has been associated with later development of psychopathology (in interaction with an adverse environment), in particular major depressive disorder (MDD), suicide attempts, anxiety disorders, and attention-deficit and hyperactivity disorder (ADHD) [16, 19, 20]. From early on, maternal sensitivity may allay some of the negative emotionality in children showing the s allele, demonstrating the interplay with the environment [21]. The s allele (coding for a hypofunctional serotonin transporter) would impair reuptake of serotonin into the presynaptic terminal, increasing availability of this neurotransmitter in the synaptic cleft. In addition to the effect on amygdala reactivity highlighted above, it is noteworthy that the serotonergic system is closely linked with functionality of inhibitory GABAergic systems (particularly with regard to prefrontal cortex-basolateral amygdala connectivity, mediated through GABAergic intercalated cells), additionally informing how fear modulation and threat processing will take place during life [22]. Animal models of knockout groups for the SERT (in an attempt to replicate the effects of the s allele) have shown that the amygdala is primed for aversive conditioning, given the decrease in 5-HT1A sensitivity [23] and an increase in 5-HT2C expression [24], serotonin receptor subtypes which are anxiolytic and anxiogenic, respectively. It is important to underscore that these are far from negligible genetic vulnerability markers; for instance, the prevalence in the general population of the s allele of SERT is around 43% [19] and that of a hypofunctioning MAO-A allele is approximately 29% [25].

High-induction single nucleotide polymorphisms (SNPs) of the FK506 binding protein 5 (FKBP5) gene (see discussion below in Section 2.1.2) have been associated with higher glucocorticoid receptor (GR) resistance and hence greater circulating cortisol levels secondary to a reduced negative feedback loop. Given higher baseline cortisol, an individual may have more difficulty recovering from a psychosocial stressor, as well as more enduring symptoms during this recovery period [26]. Studies have associated these SNPs with a higher prevalence of MDD [27] and suicide attempts [28], both in interaction with environmental adversity. The brain-derived neurotrophic factor (BDNF) Val(66)Met carriers also show an environmentally informed change in circulating BDNF levels, with lower concentrations being found in individuals who have suffered childhood abuse [29].

The following section will discuss epigenetic modifications which can occur with adverse environmental exposure; synergistic effects of genetic along with epigenetic changes may lead to subsequent psychopathology, as will be explored further.

#### 2.1.2. Epigenetic Changes

An exhaustive review of epigenetic changes associated with early life adversity is beyond the scope of this paper. There are comprehensive reviews which can detail DNA and histone changes and nontranscribing RNAs, as well as their effects on psychopathology, in much greater detail [30, 31]. Given its focus in recent psychotherapy research, DNA methylation will be the primary point of discussion with regard to epigenetics. Gene methylation serves a repressive function, as it leads to a tighter coiling of the chromatin, decreasing expression by blocking access of transcription proteins to the specific sequences. Methylation primarily occurs on cytosine nucleotides, forming methyl-cytosine. Despite a genome-wide wave of demethylation which takes place after fertilization, a number of methyl groups still remain in the DNA by action of the methyl maintenance transferases. This allows for inheritance of epigenetic changes from parent to offspring [32], a phenomenon which can be correlated with subsequent behavioral patterns and psychopathology. Methylation per se is necessary for maintaining neuronal structural integrity; methylation enzyme knockout has been shown in animal models to cause profound intellectual disability [33]; experimental models in human embryonic stem cells show that deletion of DNA methyltransferase 1 (DNMT1) resulted in rapid cell death due to DNA damage and G1 cell cycle arrest, being thus incompatible with life [34]. In addition to its regulatory function, gene methylation can also be an epigenetic marker of trauma [35] and may affect a wide range of neuroendocrine and neurotransmitter systems, as well as informing later psychopathology. How transgenerational methylation impacts offspring is the subject of much current research effort. It has been displayed in animal models regarding fears of certain odors [36]; in the cited study, aversive conditioning to acetophenone was induced in F0 mice. Offspring of these mice demonstrated enhanced sensitivity to this odor, as indicated by an odor-potentiated startle response. There was a significant increase in populations of olfactory sensory neurons which detect this odor, as well as hypomethylation of the gene (*Olfr151*) responsible for transcribing the particular acetophenone-sensitive receptor (M71), with increased genetic and receptor expression. Another study showed that male mice exposed to unpredictable maternal separation early on later developed anxiety and depressive-like behaviors, as well as impairment in social functioning; these phenotypes were later observed in their offspring, even in circumstances of adequate maternal care [37, 38]. While human infants exposed to severe neglect have shown widespread patterns of hypermethylation detected in blood cells [39], a number of key genes involved in the regulation of the stress response and of neurogenesis can be affected by early life adversity. The following discussion will highlight some of these genes and the implications of their epigenetic modifications.

FKBP5 is a cochaperone which, along with heat shock proteins, facilitates transportation of cortisol to the nucleus, where it exerts effects on gene transcription. The function of FKBP5 is to increase the resistance of the GR to cortisol, in effect increasing levels of the latter. An excessive amount of cortisol may have a cytotoxic effect, triggering the apoptotic cascade [40]. Indeed, epigenetic methylation of the GRs (including those located in the hippocampus) and demethylation of the FKBP5 gene have been implicated in the response to stress [41], and these can lead to persistently elevated cortisol levels. Maternal stress during pregnancy can induce placental gene methylation of FKBP5 and of 11-beta-hydroxysteroid dehydrogenase 2 (the latter involved in inactivating glucocorticoids) [42]; this has an adverse impact on fetal coupling, a correlation between the fetus's heart rate and motor activity and an indicator of nervous system development. What seems to be the evolutionarily “adaptive” message from the parent to the child is that the world is a place that needs to be considered dangerous, and as such the child's neuroendocrine system is primed to react to its environment in such a way. In a study of Holocaust survivors [43], FKBP5 was shown to be hypermethylated in survivors and demethylated in their offspring. Trauma disorders can be associated with low cortisol levels, which would in theory allow for norepinephrine to be released excessively without the opposing effects of the glucocorticoid, accounting for the characteristic symptom spectrum, with heightened alertness and intrusive reexperiencing [44]. However, what is passed down to the infant is an epigenetic modification which would allow for increased cortisol from early on, again emphasizing that the child should be prepared for trauma and, seemingly reinforcing the self-kindling cycle, parental trauma has been associated with subsequent trauma in their offspring [45].

Methylation of genes impacting neurotransmitter systems has also been described. In a rat model, early life adversity can lead to methylation of the glutamic acid decarboxylase 1 promoter (GAD1) [46]; this enzyme is responsible for conversion of glutamate to gamma-amino butyric acid (GABA); thus, gene repression would lead to a relative excess of glutamate relative to GABA; glutamate serves a vital role in the normal development of synapses, and excess levels can lead to elimination of nonredundant circuits, particularly during the perinatal period. In addition, there may be methylation of genes which underlie expression of N-methyl D-aspartate (NMDA) receptor subtypes [47]; NMDA subunit switch is fundamental for consolidation of synapses and thus of long-term memory [48], and this may be impaired in the event of adverse experiences. Furthering this argument, the BDNF gene has also been shown to suffer epigenetic changes in response to childhood adversity. Given the affinity of methyl groups for cytosine, it is of particular interest that the BDNF gene is rich in cytosine-guanine (CG) islands, and methylation of this gene can persist into adulthood [49]. This can influence neurogenesis throughout the life cycle, as well as showing an association with later pathology; BDNF gene methylation has been associated with completed suicides [50] as well as with development of borderline personality disorder (BPD) [51].

Further discussion of the mother-child dyad will be presented later, in particular as it relates to neural circuitry formation and strengthening of particular networks depending on the level of attunement. Oxytocin is a neuropeptide which is involved with social affiliation, maternal behavior and trust, bonding, and ascribing salience to social cues [52]. Early life stress may lead to low levels of oxytocin in the cerebrospinal fluid (CSF) of women [53], thus potentially impairing the bonding process with her infant; rodent models have also demonstrated a decrease in oxytocin receptors (OXTR) in the brain when exposed to conditions of suboptimal nurturing [54]. Despite low levels of oxytocin and of OXTR gene expression having been linked with adverse outcomes (e.g., autistic traits and elements of psychopathy) [55, 56], they are not unidirectionally and inextricably associated with psychopathology [57, 58], and the mediating effect of the environment has been demonstrated in recent studies. Differential susceptibility may factor into this mediating effect, as higher expression of OXTRs may increase an individual's capacity for empathy [59] but may also predispose to greater sensitivity to negative environmental effects, with, for instance, higher risk for separation anxiety and disorganized attachment [60, 61]; excessive levels of oxytocin may also cause greater stress sensitivity, as there is an association with higher risk of anxiety disorders [57]. SNPs within the OXTR gene do not necessarily lead to adverse outcomes on their own and indeed may be adaptive when dealing with adverse situations (e.g., the SNP rs53576, a guanine to adenine substitution, may confer resilience in G/A and A/A individuals in the development of psychopathology when faced with stressful circumstances, as compared to G/G individuals) [62]. However, there are several SNPs (and associated CpG site methylation patterns) which can interact with abuse to increase the occurrence of anxiety and depressive symptoms (though significance is variable) [58, 61, 63]. These associations are clearly complex, and optimal levels of oxytocin are instrumental in mitigating amygdala and brainstem hyperactivity in the fear response [64]. Thus, genetic and epigenetic factors impacting its effects are of importance when considering subcortical circuitry activation in a child's development, as it is informed by the early environment and factors into later psychopathology.

### 2.2. Neural Circuitry Formation

Cortisol is produced in the zona fasciculata of the adrenal and is released as part of the adaptation to stressful circumstances, working in tandem with norepinephrine to allow for an adaptive response to a given situation. Secretion is regulated by the hypothalamus-pituitary-adrenal (HPA) axis, and circulating cortisol provides negative feedback to block proximal release of corticotropin-releasing hormone (CRH) from the hypothalamus. While the amygdala has been shown to become active very early on in life [65, 66] and conditioned positive and negative responses to stimuli occur from an early age, activation is contingent on the presence of cortisol (the amygdala is rich in GRs) [67]. Indeed, during the stress response, there is a reciprocal loop between the HPA axis and amygdala, as the latter stimulates release of CRH and can further increase cortisol levels [44]. However, prior to the amygdala becoming activated, there is a period during which the presence of the mother effectively “turns off” the GRs in the amygdala, thus blocking its activation by circulating cortisol, something which has been shown in rat pups [68] and which has applicability to humans as well. This is termed the stress “hyporesponsive” or “nonresponsive” period (here abbreviated as SHP). During the human infant's development, this corresponds roughly to the first 12 months of life [69]. This is a crucial period for attachment, and premature activation of the amygdala during this time can occur with early life stress and subsequent stimulation of the HPA axis and secretion of cortisol, as GRs can become activated when they should adaptively be blocked. Importantly, the effects of this premature activation can have long-lasting effects on the neuroendocrine response [70]. Thus, in the event of an abusive caregiver, even though non-amygdala-related attachment circuitry (much of which involves olfactory circuits and noradrenergic pathways) would draw the child closer to the caregiver, the early conditioning of the amygdala due to premature plasticity owing to elevated cortisol levels may label this caregiver as dangerous. This creates the confusing picture of attaching to destructive caregivers in a disorganized/approach-avoidance manner, a template which can become activated later in life and reenacted in adult relationships. The SHP has an interesting correlation with Erik Erikson's first stage of development, “Trust versus Mistrust” [71], lasting from zero to 12 months of age, and lining up with the child's greater acquisition of motor skills as well as the time around which the maternal “blockade” of GRs is normally lessened. At this time, conditioned responses to the environment will come more to the fore (as the amygdala is allowed to become plastic through its response to cortisol), with the child's preceding experience greatly influencing whether the template of the world to be discovered will be imbued with a predominantly positive or negative valence. It is of note that, through influence of the basolateral amygdala (BLA), memories that are high in emotional content will be encoded in a more efficient manner than emotionally neutral experiences [72], adding to the significance of how one's early affective responses are consolidated.

As a manner of providing contextual and spatial data to an individual's experiences, the hippocampus (associated primarily with episodic memory and contributing to executive functioning) can aid in decreasing activation of the amygdala [73], countering (based on experience) the primarily salience-based amygdala response. The amygdala, particularly in cases of hyperactivation when faced with stimuli which are sensed to be dangerous, can inhibit the hippocampus as well, blocking access to contextual memories which could allay fear-based responses. This is relevant, for instance, in posttraumatic stress disorder (PTSD), as the reexperiencing component heightens one's reactivity, creating an inflexible and conditioned reaction to situations removed from the original traumatic event [74], with impaired access to hippocampal contextual input which could allow for a more informed and less reactive response. From an early developmental perspective, this becomes highly relevant given the infantile amnesia phenomenon, which has posited that early memories (prior to around age four years) [75] will be erased by subsequent ones within the hippocampal circuitry (though it has been recently suggested that there may be mnemonic traces which could be subsequently activated) [76]. Given the earlier and later consolidation of amygdala and hippocampal nuclei, respectively, an individual can attribute strongly positive or negative salience to environmental components but not have access to hippocampal function as a manner of creating some “perspective” on the situation. This may be internalized as a template and default response to situations which may be very difficult to modify based on later experience, particularly if subsequent environmental factors are adverse as opposed to nurturing. Certain conditions (e.g., BPD, anxiety disorders, and MDD) which show preferential activation of the amygdala over the hippocampus may be characterized by all-or-none thinking, indicating a more crystallized conditioned response. The neural correlates of these conditions will be expanded upon later. Importantly, even when information is being integrated into the hippocampus, it may be distorted by the emotionally driven response informed by the amygdala, leading to faulty processing during information compression from the dentate gyrus to layer CA3 [77], both areas which have shown decreased volume in childhood abuse [78].

BDNF is pivotal in promoting hippocampal neuronal survival and plasticity [79]; activation of this growth factor throughout the lifespan has been linked with postmitotic neurogenesis within the hippocampal dentate gyrus. There is a close correlation between levels of glucocorticoids and BDNF, a balance which is necessary to allow for consolidation of learning [80]. However, excessive levels of glucocorticoids will lead to synaptic loss and suppress the release of BDNF, decreasing plasticity in several brain regions including the hippocampus, as the latter is rich in GRs. The importance of this lies in the negative feedback the hippocampus provides to the HPA axis, blocking release of CRH and curtailing further release of cortisol downstream. For this to happen in a balanced manner, the hippocampus needs to express sufficient GRs for this feedback loop to occur. Illuminating studies have shown that early life adversity can lead to a paucity of GRs on the hippocampus, thus hindering the feedback mechanism and leading to elevated levels of cortisol. Rodent models have shown that mothers that provide less licking and grooming (LG) to their pups will result in the latter having reduced hippocampal GR RNA (with fewer expressed receptors), elevated levels of cortisol (due to impaired negative feedback), decreased BDNF levels, and consequent reductions in hippocampal plasticity, as excess cortisol is neurotoxic, as mentioned [49, 81–84]. Thus, the destructive loop perpetuates itself, as the hippocampal neurogenesis is impaired due to high circulating cortisol, and the HPA axis is further kindled by a lack of feedback. Early life adversity has also been associated with an increase in BDNF in the BLA, promoting conditioned changes which may be more resistant to modification later in life [85]. As such, BDNF can have circuit-specific effects depending on the area involved, dampening or consolidating negative effects of stress (the reader is referred to Section 3.1. for a discussion of the prodepressant effects of BDNF release from the ventral tegmental area (VTA) to the nucleus accumbens (NAc) in chronic stress paradigms). Thus, there can be a predominance of affective or contextually driven default responses to given situations, depending on the amygdala/hippocampus balance, and the memory and affective salience are internalized in association with individuals and circumstances, generating conditioned templates, dictating how one will negotiate future experiences. There is also a functional coupling between the amygdala and the orbitofrontal cortex (OFC) [86], an area of the brain associated with reward and punishment considerations regarding a particular setting; thus, the affective salience informed by the amygdala is paramount in the approach-avoidance paradigm underlying behavioral output. This amygdala-OFC connection will create associations of neutral objects with natural reinforcing or punitive salience [87]. Much of the circuitry discussed will underlie immediate situational responses and may result in impulsivity and limited flexibility to deal with novel or complex scenarios.

**Figure 1:**
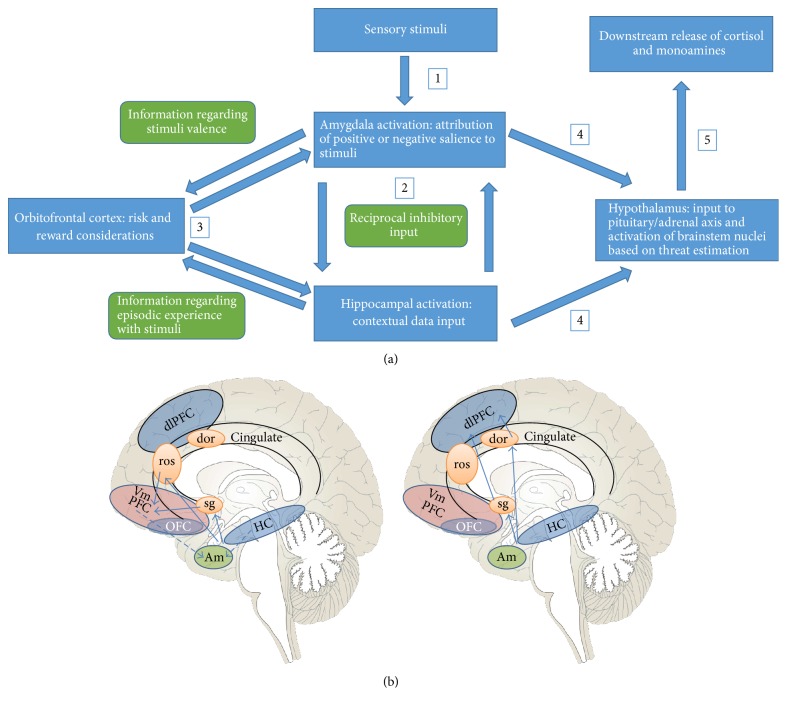
Depiction of the subcortical-cortical communication which will inform whether the dlPFC or vmPFC will be preferentially activated in response to environmental stimuli. (a) shows how, as the individual perceives an element in the environment (the thalamus is not portrayed in this figure), the amygdala will be activated, and positively or negatively valenced associations will emerge based on past experience. The hippocampus will provide some level of contextual data based on episodic memory, and the OFC will weigh risk and reward considerations based on input from these two structures. Subsequent neuroendocrine responses will ensue, with the hypothalamus being more or less driven to initiate downstream cortisol release, as well as stimulating brainstem nuclei for release of monoamines, depending on the subjective sense of danger felt to be present. (b) illustrates the subsequent higher cortical level activation that will occur after this initial communication. The subcortical-cortical connection will be mediated by the ACC (either ventral or dorsal portions), which will divert activation preferentially towards the dl or vmPFC. Left panel: in instances of lower perceived environmental threat, the vmPFC is activated via portions of the sgACC and the rACC; there is greater inhibitory connection with the amygdala, thus allowing for greater top-bottom mitigation of the fear response (also aided by the inhibitory contextual hippocampal input); more robust development of the vmPFC-amygdala and hippocampus-amygdala control mechanisms can allow for more controlled responses to the environment, even when there may be potential threat, as cognitive control and contextual data will prevent excessive reactivity and stimulus generalization, permitting greater flexibility and hence more adaptive responses. The vmPFC has been shown to be hypoactive in cases of child abuse, major depressive disorder, borderline and antisocial personality disorders, and posttraumatic stress disorder, among others (refer to text for more detail). Right panel: in situations of amygdala-driven bottom-top communication, as is seen in anxiety disorders, posttraumatic stress disorder, and borderline personality disorder, portions of the sgACC and the dACC may be preferentially activated and access the dlPFC, resulting in excessive cognitive control, attempts to suppress distressing memories, and lack of attunement with one's own emotional response, given the lack of inhibitory feedback onto the amygdala; in (b), solid lines represent excitatory connections and dashed lines, inhibitory connections. dlPFC = dorsolateral prefrontal cortex; vmPFC = ventromedial prefrontal cortex; OFC = orbitofrontal cortex; Am = amygdala; HC = hippocampus; Hy = hypothalamus; sg = subgenual anterior cingulate cortex; ros = rostral anterior cingulate cortex; dACC = dorsal anterior cingulate cortex. The sgACC has connections to both the vmPFC and the dlPFC, the implications of which are described in the text. This depiction is of the medial surface of the brain; the dlPFC and portions of the OFC are located on the superolateral surface of the cerebrum, and their representations here are schematic.

Subcortical circuitry will connect with higher cortical areas and, depending on the hippocampus-amygdala-hypothalamus output, more “cognitive” or “affective” areas may be preferentially activated as a form of adapting to the subcortical feedback (Figure 1). The prefrontal cortex (PFC) will function to negotiate more complex social behaviors through connections with the default brain circuitry; this may be done in more or less flexible manners, depending on which cortical pathways are activated. The anterior cingulate cortex (ACC) is an important intermediary in bridging areas involved in emotional processing with those involved in more nuanced cognitive functions. It will monitor error and conflict, as well as assessing the motivational and emotional relevance of stimuli, and thus predict the potential value of rewarding or punishing situations and being able to weigh different courses of action as informed by this input [88]. As alluded to above, there are two main cortical subdivisions which can be preferentially activated depending on feedback from the ACC. The ventral (“affective”) subdivision regulates autonomic/visceral responses to stress, as well as contributing to the valence one assigns to a particular stimulus. This subdivision is composed of the rostral ACC (rACC), subgenual ACC (sgACC), and ventromedial prefrontal cortex (vmPFC). The anterior portions of the ACC have connections with subcortical areas associated with instrumental responses to situations, such as the amygdala, brainstem nuclei, and periaqueductal gray (PAG); there are also connections with brain areas involved with reward and risk considerations, such as the NAc and the OFC [89]. These portions of the PFC and cingulate cortex are key in the top-down control of limbic activation and thus emotional control. Both the ACC and vmPFC have connections with the amygdala and are activated during the process of fear extinction [90–92]; due to this connection, the vmPFC can allow for a more informed cognitive appraisal of stimuli and aid in controlling the subsequent activation of the amygdala. In particular, the connection between the vmPFC and the OFC will help in this process of guiding behavior in the context of emotional input and coding one's emotional and motivational values [93, 94], which will integrate on a more conscious level the approach-avoidance templates discussed previously. Importantly, areas of the affective subdivision are also involved with empathic attunement and theory of mind (along with other areas such as the temporoparietal junction and the superior temporal sulcus) [95–97]. This will factor into later discussion regarding the effects of psychotherapy and the fundamentally interpersonal nature of therapeutic action.

The dorsal (“cognitive”) subdivision will include the dorsal ACC (dACC), the hippocampus, the supplementary motor area (SMA), the parietal cortex, and the dorsolateral PFC (dlPFC). As will be discussed, the sgACC also factors into the dorsal subdivision, which is germane in certain psychiatric disorders. While the dlPFC is involved in executive functioning, temporal information processing, the overcoming of cognitive interference, and task switching, it may also be involved in emotional avoidance and in the suppression of unwanted memories and in avoidant behaviors towards others [98]. Faulty activation may also lead to attributing excessive salience to particular thoughts and stimuli/objects, leading to perseverative and ruminative thinking and a difficulty in negotiating novelty. Thus, potentially emotional stimuli (including interactions with others) may be handled through strategies which deploy excessive cognitive control [99], as opposed to the greater cortical-subcortical connectivity described with the ventral subdivision, leading to a lack of attunement with others and indeed with one's own emotional response. The dACC has a role in pain perception, response selection, emotional regulation, and the fear response. It has been implicated in a sense of social exclusion [100] and has also been associated with child abuse and PTSD [101]. In effect, there seems to be an inverse correlation between vmPFC and dACC activity [102]. Preferential activation of the dACC can facilitate expression of the fear response, as it directly projects onto the BLA, which in turn activates the central amygdala (CeA), leading to brainstem nuclei stimulation and an autonomic response. Importantly, the vmPFC is bypassed in this process; the latter has a role in decreasing amygdala hyperactivity by projecting onto GABAergic intercalated cells (ITC) in the amygdala, which will inhibit the CeA and dampen the autonomic response [102]. With increased activity of the dACC, the ability of an individual to empathize with others may be compromised, and juvenile offenders with callous-unemotional traits exhibit enhanced connectivity between the dACC and the amygdala [103]. The dACC is a pivotal area in the social feedback network, integrating the dorsal and medial PFC [104]; thus, the valence of particular interpersonal input may be more strongly negative depending on the dACC activity level. Of note, it has been suggested that this brain area would need to be activated in order to promote psychotherapeutic change, particularly in modalities which would necessitate attunement and mentalization strategies [105]. One can speak of a “lateral bias” in circuitry activation when the dorsal subdivision is predominantly activated, something which is observed in multiple psychiatric disorders [106–110], as will be discussed in detail later. Many of the conditions in question have symptom spectra in which faulty emotional processing and preferential (and many times maladaptive) activation of cortical pathways occur which inform consolidated and ruminative negative views of oneself and/or their environment. This circuitry illustrates the powerful influence that the limbic structures can have on an individual's default appraisal and behavioral responses and may perpetuate the template that certain situations cannot be thought about and processed appropriately.

#### 2.2.1. Implications for Early Life Adversity

As mentioned, the right hemisphere is instrumental in the emotional attunement between individuals, involved, for instance, in detecting facial cues in others [111]. The mother-child dyad, on a neurocircuitry level, activates multiple circuits involving positive/negative valence, empathic attunement, and reward. When a mother looks at her child, areas activated include the amygdala, OFC, and fusiform gyrus; the latter two areas have been described as forming the “neural signature” of the parental response to the child's face, being activated on the order of milliseconds (implicating that there is a reward/risk consideration that immediately happens when a mother interacts with her child) [112–114]. The attuned mother will interact in a manner which is experienced as rewarding for her and soothing for the child; indeed, appropriate release of oxytocin enhances maternal neural plasticity [115, 116]. The rewarding nature of this interaction is illustrated by mesolimbic dopamine release [117] and activation of the NAc [118]. Cortically, the mother's ability to attune to her child is dependent on activation of the mPFC which, as discussed, is involved in the mentalization circuitry; this area is activated when the child is showing signs of distress, indicating the mother's ability to understand its suffering and provide comfort [119]. Thus, the mPFC is key in an individual's ability to access his or her own emotional response in a measured manner, as well as allowing for access and understanding of the emotional states of others. Lack of ability to do this adequately can lead to feeling overwhelmed by affectively charged situations, requiring cognitive evasion strategies. This has relevance in the development and consolidation of a child's attachment schema, which begin to take shape within the first year of life. A misattuned mother may not be able to empathically connect with her child, limiting her abilities to be soothing in moments of distress, and contributing to the child's perception that such moments may not be amenable to finding comfort in another person. Misattunement in itself predicts subsequent disorganized attachment in the child [120], speaking to this internalization and “organizing” schema of how interpersonal relationships are negotiated moving forward. This can be noted when the mother looks at her child and shows signs of shame, fear, disgust, or dissociative phenomena, among others; this can have a profound impact on the child initially on a sensorimotor, autonomic, and affective level and eventually take on greater cognitive dimensions [121, 122]. Attachment schema have been shown to be transmitted transgenerationally [123, 124], and the maternal neurocircuitry template deployed in interpersonal interactions can be passed down also. The establishment of such patterns of attachment early on is particularly relevant given that early internalized representations have been shown to correlate with relational patterns displayed as an adult [125]. Speaking more specifically to the neural circuitry activated in mothers who themselves show insecure attachment and are faced with their distressed child, what is noted is predominant activation of the dorsal subdivision, in particular the dlPFC, suggesting attempts to use cognitive control strategies to handle the situation [126]. She will also show decreased activation in the ventral striatum (where the NAc is located), indicating a lack of a hedonic response, and heightened activation of the anterior insula. The insula is a complex cortical area which is involved with anticipating and experiencing negative outcomes [106], as well as being integrated into the empathic response network; its interoceptive integrative function allows for one to appreciate a “visceral” response to a stimulus, and hyperactivation may be involved in a sense of disgust and social unfairness and pain. Thus, the interaction becomes one of displeasure which needs to be avoided, something with profoundly disorganizing effects on the child. It should be noted that, given the essentially two-person dynamic of dyadic attunement, the child's response to the mother is also responsible for activating different maternal circuits depending on the feedback she receives from the child. Thus, it has been shown through a functional magnetic resonance imaging (fMRI) study that children showing more insecure attachment behaviors will activate in the mother, during crying episodes, activation of the amygdala, parahippocampal gyrus, and insula; more specifically, disorganized attachment behaviors will lead to the mother decreasing activation of areas of the ventral subdivision, including temporal areas and the sgACC [127], furthering the challenge of attuning to a child, given the negative response bias circuitry which becomes activated, perpetuating the dyadic misattunement.

#### 2.2.2. Transgenerational Transmission of “Misattuned Template”

**Figure 2:**
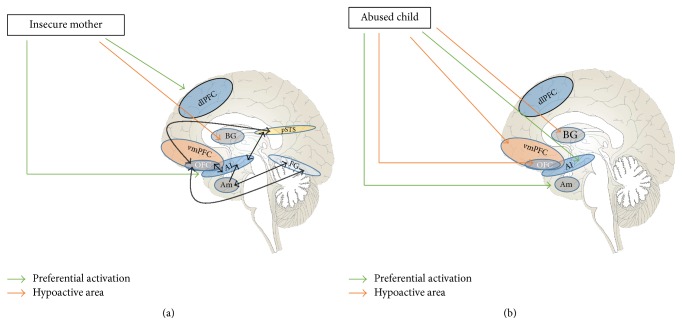
Demonstration of the transgenerational transmission of a neurocircuitry template of misattunement. (a) demonstrates the pattern observed in the insecure/misattuned mother. When looking at her child, a mother will quickly access a complex interconnected network, including facial identification areas (FG) and empathic attunement areas (vmPFC and pSTS). Importantly, in addition to these areas, there is connection with areas of the brain which are involved with immediately valenced reactions (amygdala), reward-risk considerations (OFC), and feelings of empathy versus disgust and shame (insula). Thus, there is a prominent subcortical input which will inform whether approaching one's child is something considered desirable or potentially dangerous. The misattuned mother will preferentially activate cognitive control areas (dlPFC) as opposed to the more empathically attuned vmPFC; in addition, there is heightened activation of the anterior insula (associated with social pain and unfairness) and lessened activation of areas of the VS (associated with reward to external stimuli). Thus, the model for avoidance of emotional attachment is engendered, and there is a corollary in this mother's child, which is illustrated by the CNS findings in the abused child. (b) illustrates the findings seen in a child who has suffered abuse, which seem to mirror in some important ways what was seen in the mother. Hyperactivity of the amygdala and decreased volume of the hippocampus can result in highly affectively driven responses to stimuli without access to contextual data which would allow for a less polarized reaction; thus, the greater input of the amygdala will drive the OFC balance and favor the dlPFC with regard to how the environment is negotiated; hypoactivation of the vmPFC will impair one's ability to control this amygdala response and promote fear extinction. Also, the child shows a diminished ability to see the potential positive value of rewarding stimuli due to hypoactivation of the corpus striatum, impacting how interpersonal interactions are seen, and creating a model of mistrust and negativity when dealing with other people. FG = fusiform gyrus; vmPFC = ventromedial prefrontal cortex; pSTS = posterior superior temporal sulcus; OFC = orbitofrontal cortex; dlPFC = dorsolateral prefrontal cortex; BG = basal ganglia; Am = amygdala; AI = anterior insula; HC = hippocampus. As shown in the figure, green arrows indicate greater or preferential activation, whereas orange arrows indicate the opposite. As mentioned in Figure 1, this depiction is of the medial surface of the brain; the dlPFC, portions of the OFC, pSTS, AI, and FG are located on areas of the superolateral and inferior surfaces of the cerebrum, and their representations here are schematic.

As mentioned, transmission of attachment schema from adult to child has been extensively shown in the literature.  Figure 2 illustrates several areas implicated in the maternal ability to respond to her child, and how this information is integrated into ventral and dorsal circuitry, which may cause a greater sense of connectedness or a feeling of misattunement. The “internal working model” [128] the child carries forth lends itself to a reenactment of what has been learned through his or her earliest interactions, on an affective and behavioral level. Though questions have been posited regarding the strength of the association [129, 130], abusive parenting and maternal insensitivity/misattunement may be passed down from one generation to the next [131]. In addition, abused children may grow up to display interpersonal violence in other settings (outside the parent-child dyad), as both abusive and abused parties [132, 133]. The presence of a laterally biased neurocircuitry template (largely favoring networks within the dorsal subdivision detailed earlier) has also been demonstrated in studies of individuals suffering abuse as a child, showing parallels with the circuitry discussed above for the misattuned mother. Child abuse has been associated with heightened responsiveness of the amygdala (including for neutral stimuli) and of the insula [134–136]. This shows the maladaptive template of sensed environmental danger and self-identification as an unworthy and shameful individual. There is also a hypoactivation of areas within the ventral cortical system, thus limiting the ability to control one's hyperarousal. Decreased activity in abused children has been shown in areas of the temporal lobes, hippocampus, the mPFC, the OFC, and the ventral ACC (vACC) [134, 137, 138]. The vACC hypoactivation gives way to preferential activity within the dACC [139]; thus, the child's own emotional awareness and the ability to express his or her feelings may be impaired. In addition, abused children show a decrease in their ability to anticipate and process environmental cues experienced as rewarding, as demonstrated by hypoactivation of the corpus striatum [140, 141], impairing dopaminergically driven hedonic responsiveness. This underlines how interpersonal reactions are experienced as unrewarding and potentially dangerous. Corollaries can be found in animal models; for instance, maternal separation in rodents can lead to decreased sensitivity to opioids [142]; also, chronic social defeat can lead to a decrease in affinity for natural rewards (e.g., sucrose solution), which is a model for anhedonia and can develop conditioned place preference (CPP) for low doses of cocaine (insufficient to produce CPP in controls), showing a preference for more strongly reinforcing substances to achieve a hedonic response [143]; these two examples may have translational relevance regarding the cooccurrence of psychiatric disorders with substance use.  Figure 2 outlines the transgenerational neural template of abusive or misattuned parent-child dyads.

#### 2.2.3. Lateral Circuitry Bias in Psychopathology

Establishment of an early neural circuitry favoring the cognitive/dorsal subdivision (with particular emphasis on the dlPFC), in lieu of the affective/ventral one, has been shown to carry over into later life and may inform subsequent psychopathology, as there are similar patterns seen in a number of major psychiatric illnesses (e.g., anxiety disorders, MDD, PTSD, and borderline and antisocial personality disorders). In Section 4, the pertinent neurobiology relating to these disorders will be reviewed, as will the effects of psychotherapy on these circuits.

## 3. Environmental Input and Effects of Psychotherapy

This section will expand upon the genetic and circuitry concepts discussed thus far. Particularly, the ever-dynamic interaction between the environment and the established epigenetic and neural footprints will be discussed and how continued input can effect change throughout the course of one's life, for better or for worse, depending on how nurturing or challenging circumstances are. It is known that EE has been associated with hippocampal neurogenesis, particularly in the dentate gyrus [150, 151]; there is also a demonstrable effect of EE on synaptogenesis and dendritic branching [152]. In keeping with the concept of the importance of continued environmental input in the face of plastic changes, it should be noted that new hippocampal neurons require at least a two-week period to mature before being able to contribute to cognitive functioning [153], underlining the susceptibility during this period and the need to take the notion of neurogenesis cum grano salis. This is why facilitating gene plasticity (e.g., BDNF gene) through demethylation or creating new neurons is not an inexorably positive change, and this is where the concept of “differential susceptibility” becomes key, as will be discussed.

### 3.1. Gene Plasticity and Epigenetic Influence

DNA methylation, histone acetylation, and the presence of noncoding RNAs are implicated in memory formation [153] and are the target of current research to understand the influence of EE on epigenetic markers, with consequent impact on cognitive processes [154]. Environmental stimuli can lead to neuronal plasticity by dynamically inducing chromatin modifications [155].

As mentioned earlier, VTA-NAc BDNF release can have a prodepressant effect in chronic stress. A study by Wook Koo et al., employing a 10-day chronic social defeat stress (CSDS) protocol in mice, showed that BDNF signaling in the NAc is the primary mediator of CSDS-induced social avoidance (as opposed to dopamine) [156]. This was supported by demonstrating an exacerbation of social avoidance via phasic optogenetic stimulation of the VTA after the defeat episodes. In addition, intra-NAc infusion of a BDNF tyrosine receptor kinase B (TrkB) inhibitor blocked the social avoidance induced by CSDS [156], as does local* BDNF* gene knockdown in the VTA [156, 157]. The relevance of this function factors into a model of resilience to social stress. Resilience itself is an active process [158], and individuals demonstrating greater tolerance to adverse environmental stimuli have shown adaptive gene expression which quantitatively can surpass the expression of the more susceptible organisms. A study by Krishnan et al. demonstrated that mice which were susceptible to a social defeat model of depression showed much less of a genetic modulation as compared to the resilient group (the latter showing greater time spent in an interaction zone with a social target, as well as increased sucrose preference, despite being exposed to the same social defeat paradigm). More specifically, the resilient mice showed increased gene expression with an upregulation of the number of membrane potassium channels in the presynaptic neurons projecting from the VTA to the NAc, with a diminished release of BDNF, results which suggest a decrease in the synaptic consolidation of the adverse response to the defeat model [143]. This defeat model exemplifies a level of continued negotiation of the environment in a more adaptive way, as opposed to adopting a passive response and avoidance after social defeat, a difference which may carry evolutionary implications.

The impact of the environment cannot be viewed only through the lens of the resilience-susceptibility dichotomy. This has been shown in animal models in which the experimental group has been specifically bred to demonstrate a depressive phenotype. The study by Mehta-Raghavan used the Wistar Kyoto strain of rats and bred them to display susceptibility genes implicating 14 transcriptomic markers which have been associated with MDD in humans; these genes are involved in such processes as neurodegeneration, synaptogenesis, neuronal migration, and hippocampal excitability [159]. The susceptible and control group (termed “more immobile”: WMI, and “less immobile”: WLI, respectively, depending on how they performed in the forced swim test) were then either submitted to no intervention, chronic restraint stress (CRS), or to EE. With regard to EE, the WMI group showed improvement on the FST, and subsequent mobility levels did not statistically separate from the WLI that had no active intervention. This indicates the potentially corrective environmental impact despite prominent genetic susceptibility.

The translational value of animal models is intriguing and will require further elucidation with regard to its applicability in humans. However, it seems incontestable that one's genetic make-up is not entirely deterministic, and the individual's environment will continue to shape gene expression over time. This paper posits that psychotherapy serves as a positive environmental input (something akin to EE). The split model of psychotherapy and medication management, something which is becoming more mainstream within psychiatric practice, indicates that there is a structural dissociation within the treatment model which may not be grounded on where the evidence base is leading. Many patients end up only choosing to take medications and not engage in therapy. Even those who do psychotherapy (which may be limited to very few and irregular encounters) may be immersed in an environment outside the treatment setting which may be quite detrimental and much more impactful than the work the therapist is trying to carry out. As mentioned, even with newly generated neurons, there is a “labile period” during which the continued input of the environment will inform whether or not adaptive or maladaptive memory reconsolidation will occur; in the case of the latter, already existing dysfunctional behavior may be exacerbated [160]. It is known that antidepressants can lead to BDNF demethylation, thus allowing for greater gene expression and hippocampal plasticity [161–163]. However, the notion that medications can promote “gene plasticity” (i.e., greater responsiveness to the environment, be it positive or negative), as opposed to unidirectionally leading to adaptive changes, needs to be considered in light of the split model. Two studies in particular have highlighted this in rodent models [164, 165]. Chronically stressed mice were given long-term treatment with fluoxetine and then exposed to either EE or a continued stressful environment. In the EE group, there was a decrease in depression-like behaviors, an increase in hippocampal BDNF, and a decrease in corticosterone. In the adverse environment group, despite being on fluoxetine, there was a worsening in the depressive symptoms, lower BDNF levels in the hippocampus, and higher corticosterone levels as compared with before fluoxetine was introduced. Thus, as has been suggested before [166], it is more important to consider genes as plastic as opposed to seeing them in a dichotomous manner. The human corollaries may be expanded upon through further research. The STAR^*∗*^D Trial (Sequenced Treatment Alternatives to Relieve Depression) indicated that there are environmental factors which contributed to a positive response to citalopram in humans (namely, income and employment status) [167]. Children and adolescents who have experienced early environmental disruption show a poorer response to fluoxetine, while those with greater measures of family functioning have shown greater rates of response [168]. An interesting parallel can be seen with D-cycloserine (DCS), an NMDA agonist which can be used as an augmentation strategy for cognitive-behavioral as well as exposure therapy for anxiety and trauma- and stressor-related disorders [169]. DCS can increase NMDA plasticity and accelerate responses to treatment; however, this can facilitate extinction or* enhance* consolidation of fear memories, depending on the success of the treatment [170, 171]; thus, the patient can clinically worsen as a result of this adjunct in suboptimal treatment settings.

This notion of genetic plasticity is at the core of differential susceptibility. To highlight this, two of the studies mentioned above will be discussed, both by Caspi et al. [15, 16]. In the study investigating the role of hypofunctioning MAO-A alleles, the negative outcomes (antisocial personality disorder, conduct disorder, conviction for violent offenses, and disposition toward violence) only occurred in the event of early life adversity [15]. When looking at the group that did not suffer maltreatment, the studied outcomes pertaining to the spectrum of antisocial behavior and personality traits were actually found to be* less* than the group with the normally functioning alleles. Thus, the excess of neurotransmitters conferred by the hypoactive MAO-A proved to be more adaptive in optimal settings. While an excess of monoamines can be associated with psychopathology, increased levels of noradrenaline and dopamine can also be associated, given the proper environment, with prosocial and egalitarian behaviors, as well as with cognitive flexibility [172, 173], all of which are diametrically opposed to typical antisocial traits. A similar phenomenon has been described with regard to predisposition to ADHD and MAO-A levels [174]. In a similar vein, individuals homozygous for the 5-HTTLPR s allele have shown more adaptive measures of social and mental health functioning under nurturing conditions, as compared with individuals with the l allele. Serotonin, as mentioned, is involved with encoding of fear/threat processing; under optimal conditions, this neurotransmitter will perform this function in tandem with GABA and allow for adaptive responses to the environment without an excessive arousal of the fear network [22]. This is applicable to depressive symptoms [175, 176], anxiety [176], and ADHD [20]. Thus, how neurotransmitters are deployed will be contingent on more permissive or limiting environmental factors. An example of this would be that individuals with anxiety disorders homozygous for the s allele may be more responsive to cognitive-behavioral therapy (CBT) [177], showing a greater ability to benefit from the environmental modifier offered; however, this finding has been challenged by some authors [178, 179].

Though no studies as to the knowledge of this author have been conducted looking at the effects of psychotherapy in interaction with genes implicated in anticipation and processing of rewarding stimuli, they may be a target for future research. As mentioned earlier, early life adversity can be associated with decrease in reward sensitivity; however, distinct patterns have been described with respect to the two stages (i.e., anticipating and processing rewards). Functional imaging studies have shown a decrease in basal ganglia (BG) and ventral striatum (VS) responses during reward anticipation [141, 180], but a greater response in these areas during delivery of the reward [181]. Within the spectrum of psychopathology, control individuals with impulsive traits [182], as well as individuals with psychiatric diagnoses predisposing them to impulsive actions, such as ADHD, have shown heightened reward sensitivity and VS response to incentives [183, 184]. Studies have aimed to understand the effects of adversity on individuals with particular gene variations. The catechol-O-methyl transferase (COMT) enzyme is involved in the breakdown of catecholamines. The Val158Met polymorphism is characterized by a replacement of valine by methionine at codon 158 on chromosome 22q11.21. Individuals homozygous for the Met allele display a decrease in COMT function, with resultant higher synaptic PFC dopamine levels [185, 186]. This can result in limited flexibility in processing rewarding stimuli. Indeed, Met homozygotes have been shown to display increased activation of the PFC and VS when anticipating rewards [187, 188] (though another study only found increased PFC activation) [189]. In instances of stress during childhood, Met homozygotes displayed ACC and VS hyperactivity at reward delivery, highlighting the gene versus environment (GxE) implications of this polymorphism [190, 191]. Given the heightened reward responsiveness, studies have extended to investigate whether the association ties into psychopathology; despite no clear association emerging between COMT and illicit substance use per se (with the possible exception of tobacco use disorder) [192], increased VS activation for Met carriers parallels the activation noted in the VS in substance users compared to controls when positive incentives were provided [193]. Expression of this allele, in combination with childhood adversity, may lead to a heightened pleasurable experience when under the influence of substances, potentially increasing the risk of subsequent use disorders [190, 191, 194]. Curiously, there is a decrease in reward anticipation and increase in reward responsiveness in adolescents, with the effects of the polymorphism showing a parallel with this population (which seems to have more hedonically drive and consummatory behaviors) [195, 196].

The dopamine receptor 4 (DRD4) is part of the D2-like family of dopamine receptors and contains seven transmembrane domains; it has been shown to be present in the mesocorticolimbic pathway, being thus implicated in cue reactivity and in the reward pathway [197]. The DRD4 variable number of tandem repeats (VNTR) polymorphism (exon 3) has also been a subject of attention with regard to GxE interactions as they pertain to the reward system. This VNTR is suggested to be implicated in downstream changes in cyclic adenosine monophosphate (cAMP) expression [198]; more specifically, the long (L) allele is associated with a decrease in ligand binding and reduced cAMP formation when dopamine binds to the receptor [198, 199]. The L allele is thus associated with a decrease in mesocorticolimbic dopaminergic transmission, resulting in increased craving and arousal to cues relating to potential rewards [197, 200]. These individuals have shown, when raised in conditions of socioeconomic disadvantage, to prefer smaller immediate rewards over those that are larger and more delayed, highlighting the interaction with adverse environmental influences as it relates to reward sensitivity; the differential susceptibility factor has been highlighted by the greater appreciation of future rewards of people with this polymorphism in the* absence* of adversity [201].

### 3.2. Epigenetic Changes Observed with Psychotherapy

Given the established importance of continued environmental input on epigenetic modifications, research has begun to emerge on such changes as they relate to the effects of psychotherapeutic interventions. Some of the genes discussed previously in this paper have been the target of these research efforts, and these studies will be reviewed in more detail. Importantly, while some forms of psychotherapy will be mentioned in this section, they will be elaborated on in greater detail in Section 4.

Methylation of the BDNF gene has been associated with BPD [202], particularly given this disorder's high rate of childhood abuse. Dialectical behavioral therapy (DBT) has a strong evidence base for treating this condition, and the effects of DBT on BDNF gene methylation have been assessed (DBT will be described in greater detail later) [51]. In this study, 115 patients were assessed after four weeks of DBT. Nonresponders showed an increase in methylation of the BDNF gene exons I and IV (as assessed by bisulfite treatment; DNA was extracted from leukocytes), while responders showed a decrease in methylation; these results held when adjusted for medication effects (all patients in the study were medicated concurrently). Decreases in methylation correlated with symptomatic improvement (depressive symptoms, hopelessness, and impulsivity); on the depression scale, only those with greater than 70% improvement (as assessed by the Beck Depression Inventory II) showed a statistically significant decrease in methylation.

Prolonged exposure (PE) is an intensive structured psychotherapy utilized for PTSD [203] (refer to description later in this paper). One study looked at the predictive value of GR NR3C1 (exon 1F) and FKBP5 methylation on the response to 12 weeks of PE in patients with PTSD [204], as well as the response-methylation correlation after treatment (peripheral mononuclear cells were utilized). Higher pretreatment levels of GR methylation were associated with greater response to PE. In contrast, there was no clear predictive value of pretreatment FKBP5 methylation on response outcomes. Treatment responders showed a negative correlation between GR 1F methylation and self-reported PTSD symptoms. Conversely, they displayed a correlation between decreased FKBP5 methylation and lower scores on the Clinician-Assisted PTSD Scale (CAPS) after treatment. Both of these epigenetic changes correlated with an elevation in cortisol levels posttreatment. It has been demonstrated that clinical response to brief eclectic psychotherapy in patients with PTSD can lead to an increase in cortisol levels [205], showing a neuroendocrine parallel with the epigenetic changes outlined. While improvement in symptomatology may be associated with a decrease in cortisol reactivity [206] (high reactive levels being associated with consolidation of traumatic memories) [207], higher baseline levels of cortisol may be more adaptive in PTSD given the mitigating effect of cortisol on release of norepinephrine (NE) from the locus coeruleus.

In a study employing CBT to treat panic disorder, MAO-A gene methylation was analyzed (in blood cells) with regard to treatment response [208]. Panic disorder has been associated with hypomethylation (hence greater expression) of this gene [209], with an inverse correlation found between methylation and symptom severity [208]. In the Ziegler et al. study [208], after six weeks of therapy, responders were shown to have increased methylation of the MAO-A gene (reaching levels similar to controls), while nonresponders showed further demethylation. From a neurobiological standpoint, the excess of serotonin which would result from increased methylation of MAO-A would serve to decrease the activation of areas of the brain involved in the avoidance and fear responses, as well as in the heightened autonomic response characteristic of panic attacks, such as the dorsal PAG [210]; this would allow for more rostral areas (e.g., PFC, septum-hippocampus, and amygdala) to be activated and facilitate a more adaptive response [211].

Expanding the discussion surrounding SERT and epigenetic modifications, a study was conducted to assess how CBT would affect transporter methylation in children with anxiety disorders (buccal swabs were performed for DNA collection) [212]. SERT methylation has been associated with adverse life events, including child abuse [213, 214] and, as mentioned, expression of this transporter (with its consequent impact on serotonin availability) can impact how responsive an individual will be to psychological treatments [177]. Most patients in the Roberts et al. study were diagnosed with generalized anxiety disorder (GAD); responders showed a nonstatistically significant increase in SERT methylation, while nonresponders showed a significant decrease in methylation (*p* = 0.037). Interestingly, the authors conducted a follow-up assessment six months after the treatment was completed, and there was a significant increase in methylation for participants that continued to show symptom improvement during this time period (an indicator of consolidated learning) as compared to those that showed no improvement or worsened (*p* = 0.003). Thus, at follow-up, there was a significant difference in SERT methylation between responders (increase) and nonresponders (decrease). The parallel traced is between a higher SERT DNA methylation (which replicates the s allele of the 5-HTTLPR) and greater responsiveness to treatment; the lower the expression of the gene, the greater the individual would be able to respond to the positive environmental input, and the epigenetic modifications seen with successful treatment would seem to facilitate this potential for continued improvement.

Controversy remains about the value of measuring methylation changes in specific genes as an indicator of treatment response; it has been questioned what the value is of assessing methylation in peripheral tissue. It has been challenging to demonstrate direct correlations between peripheral blood cell and brain methylation [208, 209]. However, MAO-A gene methylation in leukocytes has been shown to be inversely correlated with MAO-A levels in the brain [208, 215], highlighting the utility of this measurement. Also, authors have argued that, with regard to buccal tissue, the lower cell heterogeneity and a degree of developmental commonality with brain tissue would make it a promising option for methylation studies [216]. This burgeoning field will hopefully continue to yield elucidating data.

## 4. Changing Default Neural Circuitry Activation

One change that can be fostered by psychotherapy is modification of default activation patterns involving the circuitry described before. Resting-state functional connectivity and task-related functional imaging have increased understanding of how particular areas of the brain can maintain maladaptive modes of thinking and behaving, as well as the implications of medication and psychotherapeutic treatment strategies in reconfiguring connectivity in tandem with clinical improvement or lack thereof. Dorsal or ventral bias can inform more or less functional connectivity with subcortical areas and also underlie the degree of flexibility that can be elicited when interacting with particular stimuli. Though the dlPFC may be anomalously and excessively activated in maladaptive cognitive appraisal, it is, along with the hippocampus, also one of the key areas of the brain that will need to be accessed to allow for psychotherapy to be effective [217], given that the content of one's ruminations and fixations need to be allowed room for exploration in order to promote flexibility and for an understanding of one's preoccupations to be processed and worked through. This would potentially allow for a greater fluidity between emotional appraisal and regulation, the latter implicating ventral structures (including the vmPFC), which in itself allows for a more measured control of subcortical activation [99, 218]. The vmPFC is paramount in the therapeutic response, given evidence that only engaging dlPFC and other lateral PFC regions may not be sufficient to mitigate amygdala reactivity to negative stimuli [219]. The hippocampus will allow for contextual consolidation of the response to treatment. Importantly, certain activation patterns, though involved in particular symptom spectra, may also be prognostic indicators of a positive response to treatment, as engaging these areas while working therapeutically may help to reconfigure their role within the neural circuit. For instance, ability to engage the hippocampus, which gauges the potential to provide greater contextual input to counter generalizations in belief and response patterns, has been shown to predict treatment response to CBT in adults with panic disorder (PD) and GAD [220]. The neuroimaging literature of psychotherapy will be reviewed in this section, focusing mainly on the effects of psychotherapy on conditions in which bias favoring the dorsal system is well established (anxiety disorders, MDD, PTSD, and BPD). A brief review of neurobiology will be provided prior to describing the effects of different forms of psychotherapy. Tables 1–3 provide a synthesis of the studies outlined.

### 4.1. Neurobiology of Conditions with Lateral/Dorsal Bias

#### 4.1.1. Anxiety Disorders

In the setting of anxiety, there is a bias in an individual's interpretation of the environment [107, 221], with a heightened perception of threat, a decreased recruitment of the PFC during emotionally regulated tasks [222], and an increase in amygdala and anterior insula responsiveness. Consequently, there is a difficulty in the generation of different outlooks on a given situation [223], thus the maladaptive response feeds into itself, and the “bottom-up” response is maintained [224]. Altered patterns of functional connectivity occur in corticocortical and cortical-subcortical networks (e.g., increased amygdala-dACC and decreased mPFC-OFC connectivity) [211, 225, 226], in tandem with preferred activation of cortical areas favoring rumination, negative affective cognitions, and decreased cognitive appraisal (e.g., dlPFC and inferior frontal gyrus (IFG)) [218, 227–230]. As a result, the anxious individual has an attentional bias and difficulty disengaging from threatening stimuli, magnifying the cognitive inflexibility and leading to overgeneralization [228]. This is seen pervasively in GAD, which is associated with persistent anxiety symptoms, leading to autonomic arousal and diffuse preoccupations and ruminating about numerous situations in an individual's life, without necessarily being attached to circumscribed cues, as in phobias. Excessive attention to social cues and rumination about environmental stimuli, as seen in social anxiety disorder (SAD), have also been associated with increased connectivity between the amygdala and more dorsal areas of the PFC, with a concurrent decrease in amygdala-vmPFC connectivity [231, 232]. As stimuli become more proximal, activation of fear-system components will move from lateral to central amygdala, with subsequent activation of PAG and brainstem nuclei, inducing a more pronounced panic response [233]. Of note, phobic disorders and PD are variably associated with increased activity in the CeA, insula, and PAG [234–236].

#### 4.1.2. Major Depressive Disorder

MDD has been associated with greater activity in areas of the brain associated with the processing of emotionally salient stimuli, including the amygdala and sgACC [237–240]. The amygdala may also lose its ability to discriminate between neutral and emotional stimuli [241]. Decreased volumes in the hippocampus, BG, and in the OFC have also been demonstrated [242]. The dlPFC can show increased or decreased activation, depending on the function which is being assessed. With the ruminative and poor self-view aspects, as well as the negativity bias toward external stimuli, there is an increase in dlPFC activity [108, 241, 243, 244]. This is compounded by the concurrent vmPFC hypoactivation [109, 241], which contributes to difficulty with reappraisal of negative thoughts. However, the dlPFC is underrecruited when the tasks involve frame shifting and inhibition, executive control, and planning [222, 245, 246]. There may also be differences between right and left dlPFC activation in MDD depending on the affective valence of decision-making processes [247]. Decrease in frontostriatal responsiveness to reward has been described; this includes decreased anticipation and enjoyment of rewarding objects/stimuli, as well as a bias towards overestimating failure [248–250]. In MDD, there is also difficulty in maintaining activation in the NAc during attempts to consciously upregulate positive emotions [251].

#### 4.1.3. Posttraumatic Stress Disorder

In PTSD, one of the most described neurobiological findings is a decrease in the volume of the hippocampus [252]. There is an increase in activity in the amygdala (leading to kindled fear acquisition networks) and in the insula [253], as well as an increase in hippocampal activity when encoding negatively valenced stimuli [254]. Sensitization of the dACC is associated with an increase in the appraisal of threat and expression of fear and correlates with the heightened activation of the insula and amygdala [101, 255]. Difficulty with modifying this response pattern derives from hypoactivation of the rACC, vmPFC and of the hippocampus during fear extinction [255].

#### 4.1.4. Borderline Personality Disorder

Cluster B personality disorders, in particular BPD and antisocial personality disorder (ASPD), are associated with heightened affective responses to the environment with behavioral overswings without PFC modulation; it is important to highlight that, in the case of ASPD, this applies to cases more commonly associated with child abuse and in which violence is reactive as opposed to proactive, the latter being associated with psychopathic traits, and thus with different patterns of emotional reactivity and autonomic arousal [256, 257]. Both of these personality disorders have been associated with heightened amygdala reactivity, as well as a decrease in vmPFC, reflecting the difficulty in identifying emotional states in others that are not inflexibly perceived as menacing [257–259]. Also, in BPD there is enhanced connectivity between the dACC and the amygdala and insula, heightening focus on social cues with individually ascribed salience [260].

In the following section, different forms of psychotherapy and their effects on the described circuits will be described in greater detail.

### 4.2. Psychotherapeutic Modalities and Effects on Neurocircuitry

#### 4.2.1. Cognitive-Behavioral Therapy (CBT)

CBT is a typically time-limited form of treatment which involves cognitive distortion analysis (including overgeneralizing and catastrophic thinking), situational reappraisal, exposure hierarchy analysis, and several exposure components. It has a strong evidence base for numerous psychiatric conditions, including various anxiety disorders as well as MDD [261]. Tasks utilized to assess efficacy of CBT attempt to replicate stimuli within an individual's life which may elicit more generalized or specific fear-based responses. It is at the core of CBT to attempt a reworking of cognitive schema, accessing more flexible cortical areas which may allay conditioned responses through inhibitory connections to subcortical areas. Tables 1(a), 1(b), and 1(c) detail the neuroimaging studies assessing neural markers of treatment response.

*(1) CBT for Anxiety Disorders*. CBT can address both the more ruminative/cognitive-based symptoms of anxiety disorders (as generated by diminished activation in medial PFC and areas of the ACC, with ineffective control of amygdala responses) as well as the more proximal fear-based responses (as outlined for phobias and panic disorder). Several studies have aimed at assessing the PFC-amygdala activation patterns in response to treatment; this has been done through subject responses to emotional faces [262–264]; posttreatment effects demonstrated a decrease in activation of the amygdala [262, 263], as well as within the sgACC (which can inform dlPFC activity) [263], with more ventrally favored pathways being recruited; one study showed posttreatment activation of the ventrolateral PFC (vlPFC) when exposed to angry faces (this area also aids in top-down control of the amygdala) [264]. Stimulus processing in anxiety disorders can be excessively influenced by amygdala input; CBT, through response pattern recognition and gradual incorporation of contextual data into one's appraisal of the environment, increases hippocampal activation, allowing for a more nuanced and cognitively informed processing to take place [265].

SAD can be considered a condition which factors in both the cognitive/distal and subcortical/proximal elements of anxiety and fear responses, as the reality of exposure occurs alongside pronounced ruminative processing. The importance of the cognitive component is of particular relevance when reviewing results in the literature for the efficacy of CBT, as there have been some varying findings, particularly pertaining to subcortical elements. Attenuation of enhanced dACC-amygdala and dorsomedial PFC- (dmPFC-) amygdala connectivity [226] was demonstrated in one CBT study, suggesting a more flexible top-down control. In contrast, another study showed that amygdala reactivity to negative self-beliefs remained consistent over time in the treatment group [266]; there was an increase in areas of the dorsal PFC (namely, dmPFC and dlPFC). The authors stressed the importance of these areas specifically in cognitive reappraisal, as opposed to areas of the brain involved in fear extinction. Another individual CBT study assessed the effects of treatment on emotional responsivity to social evaluation [267]. Interestingly, as with the study before, improvement in negative emotions when reappraising social criticism was associated with activation in mainly cognitive as opposed to more effectively attuned cortical areas as a result of the treatment. Increased hippocampal activation and a decrease in dysfunctional IFG-hippocampal connectivity was noted with therapist-guided CBT in panic disorder patients (as compared with self-guided treatment), suggesting cognitive reframing and increased contingency encoding may be facilitated by the therapeutic dyad [144].

With regard to more proximal fear response patterns, CBT has been shown to decrease limbic, paralimbic, and PAG hyperactivation in a public speech task, with maintenance of effects at one-year follow-up [236]; when exposed to a specific phobic stimulus (e.g., a spider), CBT can additionally promote a decrease in dlPFC and insula activation, highlighting the decrease in autonomic fear systems as well as activity in areas implicated in disgust and shame. A decrease in insula activity during fear processing was supported by an additional study of spider phobia in patients undergoing group CBT with an exposure component [268]. In the treatment of PD, CBT has been shown to decrease activation within fear-based networks (e.g., amygdala, anterior insulae, and dACC), as well as promoting more harmonious circuit connectivity. The latter is exemplified by normalization of hyperactivation in the IFG [230], as well as greater connectivity between the latter and the amygdala, hippocampus, ACC, and medial and lateral PFC, informing a more affectively informed and less cognitively controlled circuitry. In the cited study, treatment also resulted in a decrease in activation of the vmPFC, which may suggest a more integrated form of decreasing subcortical hyperactivity by deploying other areas of the extinction circuitry.

*(2) CBT for Major Depressive Disorder*. CBT is arguably the most well-validated form of psychotherapy for MDD (showing potential effectiveness over 50% of cases) [261], though evidence has supported combination treatment (with either medication or other modalities of therapy) [269, 270]. There are many studies which have gauged predictive and outcome findings relating to psychotherapy for MDD, and the effects of CBT on neural circuits in MDD are multifaceted and still an area of active research. There is some level of controversy surrounding how activity in given areas fits into the framework of this condition, and this will be discussed below.

As mentioned, changes in emotional stimulus discrimination in the amygdala and portions of the ACC, decreased input from the hippocampus, alterations in reward processing, and biased PFC recruitment are relevant in the clinical presentation of MDD. As such, the patterns of PFC, cingulate, and amygdala-hippocampal activation are the prime regions of interest throughout the studies analyzed. As noted in anxiety disorders, CBT can result in a strengthening of contextual appreciation of stimuli, with normalization of the amygdala-hippocampus activation pattern and less amygdala hyperactivity when exposed to emotional faces [240] (though it is important to highlight some level of functional heterogeneity within the amygdala, as evinced by therapeutic response leading to heightened activity to implicit processing of emotions) [271]. Modification of activation patterns with CBT also occurs in other areas involved with processing of emotionally salient stimuli, such as the precuneus and inferior parietal lobule [272]. The trend towards activating more ventral portions of the cingulate and PFC has also been demonstrated with CBT. One PET study comparing CBT to venlafaxine showed that treatment response correlated with a decrease in dorsal areas of the cingulate cortex (namely, the PCC) and with a metabolic increase in the anterior portion of the sgACC and vmPFC [273], indicating greater top-down regulatory function (this is particularly relevant given that activity in the sgACC has been associated with depression severity, as this area in MDD can show decreased volume) [274]. The increase in mPFC activity has been replicated elsewhere [241, 275]. Response to treatment has also been associated with a decrease in metabolism in the OFC [273, 276], which may indicate an increase in flexibility in terms of the fixity of salience ascribed to stimuli, given that the OFC can contribute to establishing affectively biased perceptions [273]; in line with this, it has been hypothesized that the increase in OFC activity in MDD is a compensatory mechanism owing to an attempt to regulate amygdala hyperactivity, given the decrease in sgACC/vmPFC regulatory mechanisms [274]. The decrease in dmPFC activity shown in this study [273] after CBT is also of relevance given that this cortical area is involved in self-referential aspects of emotional stimulus processing, incorporating elements of ascribing meaning to emotional experiences and recall of affect-laden past events, and again a decrease in activation may reflect greater cognitive and self-assessment flexibility. Other dorsal cortical areas (e.g., dlPFC) have been shown to decrease their excessive activity in response to treatment, in tandem with increased hippocampal and parahippocampal activity [276], again reinforcing the more harmonious PFC network as contextual input is allowed through revisiting ingrained thinking patterns. It should be noted that modification within cortical circuitry (without engaging limbic structures) can lead to clinical change. In one study [272], there was a significant positive correlation between symptom improvement and left precentral gyrus activity, a brain region involved in successful response inhibition, which may be impaired in MDD [277]. Interestingly, in this study, there was not an observable engagement in the amygdala, something which was felt to be due to the cognitively effortful nature of accessing and working with dysfunctional thoughts, an activity which may be more circumscribed to cortical areas; thus, this would be one instance in which the “medialization” phenomenon (given the dorsal and lateral localization of the structure) and greater cortical-subcortical connectivity would be bypassed, though still with clinically relevant effects.

It does need to be emphasized that these different brain regions are not uniquely ascribed discrete functions, as our understanding of functional connectivity has evolved. As such, while certain patterns can be noted in the conditions outlined, there may be variations depending on compensatory activation of other networks. One example of such is the parahippocampal gyrus in MDD. This brain area, as the hippocampus, is associated with episodic and contextual memory, assisting as well in the associative learning of positively and negatively valenced experiences, and activation is greater with novel as opposed to familiar tasks. Increased activity in this area in response to treatment may indicate incorporation of new information and modes of thinking, linking contextual input with new modes of understanding one's cognitive schemas [276]. Parahippocampal activation can also be associated with dysfunctional attitudes and negative beliefs; thus, in reworking these through CBT, activity can be decreased in this area over time [272]. Sankar et al. posited that this occurred due to a decrease in extreme negative thoughts in the treatment group as well as an increased familiarity with the material being accessed (it is of note that a similar decrease occurred in the control group, indicating this gradual assimilation of material and the lessening of parahippocampal activity is functional) [272]. Another example relates to increased activity of dorsal areas of the cingulate in response to treatment, such as the dACC and PCC (the latter also having reciprocal connections with the dlPFC; the reader is referred to the section on interpersonal psychotherapy for discussion of increased activation of the PCC) [276, 278, 279]; this finding may underscore activation of dorsal PFC regions during CBT in order to access and revisit more inflexible thinking patterns; thus, there is not uniformity in the finding of decreased dACC activity with successful treatment, though again this may be adaptive.

With regard to the reward pathway, it has been mentioned that patients with MDD have difficulty predicting positive outcomes to potentially rewarding situations. One adolescent study of group CBT assessed response to treatment through its effects on a reward paradigm [280]. Successful treatment resulted in a decrease in bilateral amygdala, hippocampus, and sgACC activity. The authors associated the sgACC hyperactivity with predicting treatment response (see further discussion regarding neural predictors of response later in this paper) and emphasized its role in erratic processing of stimuli, which may lead to poor self-views and impaired ability to appreciate positive stimuli. Behavioral activation therapy has also resulted in greater engagement of the VS and precentral gyrus during anticipation of rewards and of the OFC during reward feedback [281].

*(3) CBT for Posttraumatic Stress Disorder*. The revisiting of traumatically informed cognitive beliefs can be done through multiple therapeutic modalities (see further discussion below), many of which incorporate elements of CBT. The neurobiology of PTSD is marked by heightened activation of the amygdala-dACC network, as well as of the insula, with little room for contextual hippocampal input. Successful CBT has been shown to effect a decrease in hyperactivation within the amygdala [282], dACC, and anterior insula [283], allowing for enhanced cognitive appraisal.

*(4) Dialectical Behavioral Therapy (DBT)*. Both BPD and ASPD, as mentioned, are associated with decreased functioning of the vmPFC, interfering with empathic attunement and ability to safely regulate affective arousal when confronted with social cues. DBT is an integrative approach which has been gaining evidence in the treatment of BPD [284]; it consists of both individual and group processes and integrates elements of CBT as well as mindfulness, aiding in distress tolerance, cognitive reframing, and interpersonal skills. In addition to the neural changes deriving from cognitive reframing and working through interpersonal challenges (as described with CBT and IPT, resp.), mindfulness can be associated with increased activity with more medial portions of the PFC and ventral parts of the ACC [285], as well as a decrease in amygdala activation [286]. Neural findings in successful DBT have echoed these effects, showing increased connectivity between vmPFC and amygdala (however, this only occurred with active neurofeedback incorporated) [287] and a decrease in amygdala activation [288].

Mentalization-based therapy has been shown to be helpful in both BPD and ASPD [289] and seeks to activate the vmPFC by attempting to access the mind of the other and assess the cognitive process which unfolds with this exercise. To date, this author is unaware of neuroimaging studies which assessed brain changes with this form of treatment, but it is an exciting prospect for the future.

#### 4.2.2. Other Psychotherapeutic Modalities

This section will detail a number of different therapeutic modalities, with a brief description of the treatment, the conditions for which it is used, and the neurobiological effects seen with therapeutic success.  Table 2 describes the findings relating to these different forms of therapy.

*(1) Eye Movement Desensitization and Reprocessing (EMDR)*. EMDR has been gaining significant ground in the PTSD literature [290]. It allows for recall of difficult/traumatic images (at times with the aid of a script) while there is continued sensory input. An MRI study utilizing EMDR described the effects of 12 weeks of treatment [291]. In response to treatment, there was a bilateral volumetric increase in the hippocampus, showing enhanced ability to process traumatic memories with greater contextual input and aiding with fear extinction. The uncus (BA 36) is a portion of the parahippocampal gyrus and has a role in repetition of stimuli that have affective salience, as well as in the processing of defense responses to perceived threat; successful EMDR has been shown to decrease tracer uptake in this area, indicating downregulation of heightened responsiveness [292].

*(2) Prolonged Exposure (PE)*. PE, another treatment modality employed in PTSD [203], consists of a combination of imaginal exposure (recounting the traumatic experience) and in vivo exposure (confrontation of traumatic reminders which had been avoided); this occurs as a manner of attempting to allow cognitive restructuring and to extinguish conditioned fear responses associated with triggers. Top-down control of subcortical hyperactivity permits for fear extinction and consolidation of more adaptive associations. Two studies highlight an important contrast in terms of how this can take place in response to PE. A study utilizing cognitive restructuring with imaginal exposure utilized processing of fearful faces to gauge treatment response [293]. Decrease in CAPS scores correlated with an increase in rACC activity and a decrease in bilateral amygdala activity, demonstrating the greater inhibitory activation of this network during fear processing. Another study employing a 10-week PE protocol showed that fear extinction recall posttreatment was associated with a decrease in rACC activation and increased activity in the mPFC [294]. The rACC works in tandem with the vmPFC and sgACC in regulating the fear response; however, it has a function of monitoring external cues [295], something which can be more or less adaptively activated in individuals, depending on the surrounding structures' connection to subcortical areas, and it would seem there is not a straightforward correlation with symptom response in PTSD, given the disparate findings regarding this area in the two studies mentioned.

*(3) Brief Eclectic Psychotherapy (BEP)*. BEP includes a number of different technical elements, including imaginal exposure and cognitive restructuring, in addition to some elements of psychodynamic therapy and a farewell ritual when the treatment is complete. BEP can be utilized for treating PTSD, and there is a technical overlap with some elements of PE, given the exposure and cognitive components of the treatment. A study examining the effects of BEP on PTSD patients assessed their ability to process traumatic material as outlined in a script they prepared [253]. Successful treatment correlated with a decrease in regional cerebral blood flow (rCBF) in the right uncus (refer to the section on EMDR for functional description of this structure); there seemed to be conflicting finding regarding activation of the middle frontal gyrus (MFG; both superior and middle FG are part of the dlPFC). The authors highlighted how there are conflicting findings in the literature regarding the MFG and how greater or lower activation may depend on the script utilized. 

*(4) Interpersonal Therapy (IPT)*. IPT is a form of psychotherapy which happens in a stepwise approach to aid the individual in terms of understanding how to adapt to certain life events which may be informing their depressed mood. The main topics addressed include unresolved grief, role conflict, role transitions, and interpersonal deficiencies [296]. The studies discussed in this section will refer to management of MDD.

Retrieval of autobiographical memories may favor more dorsal areas of the cingulate cortex, and this may be a factor in the neural changes noted with IPT. There is increased activity in the dACC and PCC noted in IPT responders [145, 279], though subsequent activation of the dlPFC has been inconsistently reported [146, 279]. The PCC is an area involved in retrieval of episodic memory, and increased activity may indicate an enhanced ability to access thought and improve executive functioning [297]. As mentioned, the PCC activation pattern differs from that described by Kennedy et al. (2007) with CBT [273]; the reasons for this are not entirely clear, though the function of this area with pain perception (which may have a component of painful affect retrieval), as well as more involved discussions in IPT relating to active life role transitions with the component of grieving (as opposed to the more cognitively based technique of CBT), may have some relevance. Indeed, the PCC is involved in remembering familiar individuals in one's life as well as in the neurobiology of grief and bereavement processes [298, 299].

#### 4.2.3. Psychodynamic Psychotherapy

Psychodynamic psychotherapy is essentially a form of insight-oriented treatment, allowing patients to access origins of conflicts and understand their sense of self, defensive structures (including efforts to suppress emotional responses), and present interpersonal functioning in light of early life experience. This is integrated with an understanding of real-time dynamics with the therapist, and an appreciation of transference-countertransference factors can help illustrate the internalized relationship templates the patient may feel compelled to reenact. The latter elements are conceptually important given the empathic attunement element of the treatment (despite some level of technical variation within schools of analytic thought), as this may engage more ventral areas of the PFC and aid in decreasing maladaptive dlPFC activity as well as amygdala hyperactivity. In MDD, psychodynamic therapy promoted decreased activation of the dorsally biased bottom-up network, with mitigation of subcortical and limbic hyperactivity [300–303], a decrease in sgACC hyperactivity (an area involved in poor self-esteem, guilt, and repression of emotions) [300, 304], diminished activity in dorsal areas of the mPFC (associated with ruminative self-referential thought) [300, 305], increased activity in vPFC [302], and normalization of SERT binding within medial portions of the PFC (as noted in a SPECT study; PFC-amygdala top-down control relies on serotonergic transporter integrity) [306, 307]. Modulation of serotonergic transmission within the mPFC was shown in a PET study assessing 5-HT1A density [308]. Psychodynamic therapy was associated with greater binding of 5-HT1A in the mPFC and OFC, a finding which correlated with clinical improvement. As 5-HT1A is an inhibitory receptor, this may serve to counterbalance excessive glutamatergic tone in a number of brain regions; MDD has been associated with greater 5-HT2 receptor density in the amygdala and PFC [309], which has also been linked with suicidal behaviors. Interestingly, the authors correlated the increase in 5-HT1A binding with greater social functioning [310], replicating the notion of heightened interpersonal attunement based on experiencing an empathic other in a therapeutic setting.

Effects of psychodynamic therapy have also been studied in subjects with BPD [311]. Pretreatment imaging showed increased perfusion in limbic areas and diminished perfusion in the PFC; throughout the course of treatment, there was improvement in numerous clinical parameters (e.g., impulsivity, self-injurious behaviors, therapeutic alliance, and adaptiveness of defense mechanisms), which correlated with an increase in perfusion in the frontal cortex, reflecting enhanced limbic modulation through cognitive strategies.

Table 3 outlines the studies regarding psychodynamic psychotherapy. It should be noted that the open-endedness of treatment lends itself to some replication challenges, and there is a considerable difference in treatment length in some of the studies mentioned.

### 4.3. Neural Predictors of Treatment Response to Psychotherapy

Extending the understanding that has emerged from neural changes which occur with successful courses of psychotherapy, numerous studies have looked at activation patents within neural areas or circuits which could potentially predict an individual's response to a particular therapeutic intervention. Areas which are actively engaged during treatment may, through established pretreatment activation patterns, lead to further understanding of their role in psychopathology and treatment response. Neuroimaging could therefore be an ancillary method of tailoring treatment modalities to patients suffering from particular conditions. The reader is referred to additional reviews of the evolving use of this data in guiding treatment [312–314].  Table 4 outlines the studies investigating predictors of psychotherapeutic response.

As described in a previous section, individuals with anxiety disorders can display heightened reactivity to threat. Hyperactivity in particular brain areas involved with threat appraisal and response has been shown to positively predict response to CBT in the treatment of anxiety. This applies to activity patterns in the amygdala (both hyper- and hypoactivity have been associated with predicting response) [147, 262], as well as increased activity in the dACC, hippocampus, and areas within the temporal and frontal lobes involved with one's reaction to threatening stimuli [147, 315–317]. As these regions show changes over the course of treatment, it is hypothesized that higher levels of activation may indicate greater recruitment during interventions which require modulatory network engagement. Functional coupling between areas of the ACC and amygdala may predict whether an individual will benefit from CBT. Activating the dACC and a decreased dACC-amygdala coupling during a self-criticism task has been shown to predict sustained response to treatment one year later [318]. In addition, increased pretreatment ACC-amygdala inhibitory coupling during fear conditioning has been associated with treatment response [148, 265]; interestingly, the l allele of the 5-HTTLPR polymorphism seems to be linked with this greater coupling posttreatment and may drive treatment response (though the allele per se does not seem to predict response) [178]. Carriers of the s allele display a decrease in the ACC-amygdala connectivity [319], which may be responsible for considerable variation in temperamental anxiety traits, and theoretically confer greater resistance to therapeutic interventions. Another instance of genetic susceptibility to treatment nonresponse pertains to the 5-HT1A rs6295 polymorphism; the G allele has been associated with increased presynaptic autoreceptor expression (decreasing serotonin release due to inhibitory feedback) and decreased postsynaptic receptor expression (potentially leading to heightened glutamatergic drive and contributing to anxiety and depressive symptoms) [320]. The G/G genotype has been associated with fear generalization, heightened awareness to threatening stimuli, and greater avoidance and escape behaviors, informed primarily by the amygdala and hippocampus [149]. Resistance to CBT was shown in G/G carriers, as indicated by continued amygdala hyperactivity in response to threatening and safety cues, as well as inability to promote differential conditioning in ACC and insulae; G/G carriers also showed a decrease in self-initiated exposure behaviors, decreasing likelihood of benefitting fully from treatment [149].

In PTSD, pretreatment hyperactivity in amygdala and ACC (both ventral and dorsal) was associated with treatment nonresponse [282], which the authors hypothesized to indicate excessive affective discharge given the rapid presentation of stimuli, and question whether this is an adequate parallel with PTSD, particularly given that this finding has been challenged by other studies, which emphasize the importance of engaging the ACC for adequate treatment response. Along the same lines, a decrease in limbic and paralimbic gray matter density was a predictor of treatment response in an EMDR study [321]. As a corollary to the ability to appropriately control one's responses to environmental stimuli, PTSD subjects also showed greater response to CBT when displaying higher pretreatment activation of frontostriatal networks during inhibitory control tasks; in contrast, poor response was predicted by pretreatment requirement of a more widespread engagement of cortical and subcortical areas to perform a similar task; thus, a more contained preexisting circuitry can be strengthened by psychotherapy [322].

With regard to the predictive neuroimaging findings for the use of CBT for MDD, findings in the literature have at times been contradictory, and further research efforts seem warranted. Despite hyperactivity in the amygdala being described in MDD, this has not been a consistent region of interest (ROI) in studies assessing neurobiological change with treatment. Network remodulation on a “cortico-cortical” level may be more salient in assessing the effects of CBT for depressed patients [323], given the ruminative and negative thought patterns that emerge (different from anxiety and trauma-related disorders, in which the immediately threatening component is of prime concern and in need of attention and active cognitive reworking to promote symptom improvement). Though subsequent top-down influence will be paramount in modifying insula and amygdala-based negative salience of environment and self, predictive factors regarding treatment outcome seem to lie more within PFC and ACC patterns of activation [324], and dysfunctional PFC-ACC can be responsive to CBT [325]. Amygdala hyperactivity is mitigated with treatment, but it is not a consistent predictor of response to CBT in subjects with MDD. Greater pretreatment activation of both dL and vmPFC areas is predictor of successful treatment outcomes [241, 326]. Activity in these areas would allow for individuals to, respectively, engage in cognitive reappraisal (a function which can be impaired in MDD) [326] as well as allowing for more active vmPFC-amygdala control over the course of treatment, limiting subcortical input which could make the work overwhelming or distressing, hindering progress. There are conflicting findings in the literature with regard to predictive factors relating to activity within the different areas of the ACC, as functional divisions are not entirely clear. Within the subdivisions of the ventral portions of the ACC, the “affective” and “cognitive” functions may at times be less clear-cut, and it has been hypothesized that the pregenual cingulate is something of a watershed area [327]. This overlap can be appreciated with regard to the dlPFC and its connectivity to both rACC [Brodmann area (BA) 24] and sgACC (BA 25). Treatment nonresponse has been associated with a decrease in dlPFC metabolism and decreased activity in the rACC [273, 328]; this may reflect difficulty accessing executive functioning regions of the brain as well as top-down regulatory areas, making it difficult to engage in the process inherent to CBT, particularly working with distorted cognitive patterns. In MDD patients, the sgACC has been shown to have decreased gray matter volume and metabolic activity [329]. Low baseline sgACC activity and deficient sgACC-amygdala regulatory coupling may inform negatively ruminative thought patterns [330], and it has been hypothesized that CBT will be most useful in these cases because the inhibitory control can be enhanced through successful therapy [330]. Hyperactivity in a region between the rACC and sgACC has been associated with resistance to both CBT and antidepressant response [331, 332]. In effect, response to CBT seems to be favored in patients with a stronger rACC-dlPFC and a weaker sgACC-amygdala pretreatment activity, suggesting that revisiting maladaptive schema indeed requires active revisiting of one's cognitive distortions, and enhanced top-down control may be more important with regard to the vmPFC. However, it is not entirely clear whether circumscribed functions and responses of the sgACC can be promoted, in particular as this area can undergo, as mentioned, different activation patterns posttreatment [273, 280, 332]. Interestingly, increased activity in the sgACC may predict response to psychodynamic therapy in depressed patients (activity which lessened over the course of treatment) [300]. Interpretation of this finding is somewhat conjectural, though given the variable nature of technique in psychodynamic therapy, preemptively active engagement of sgACC-limbic networks may be more adaptive depending on the material that is revisited. In a study utilizing the Core Conflictual Relationship Theme (CCRT) manual to guide psychodynamic psychotherapy, treatment was associated with a decrease in insula activation [303]; in addition, precuneus activity prior to therapy was associated with greater psychological mindedness (associated with modest predictive value in dynamic therapy) [333]; both areas are associated with self-attributional beliefs; thus, being able to realistically access one's understanding of internal responsibility for external events (balanced between anterior and posterior portions of the precuneus) may help [334], particularly in this model of therapy, which reworks problematic interpersonal relationships. Importantly for psychodynamic therapy, the precuneus is involved in several dimensions of self-awareness, including taking the first-person perspective, episodic memory, and self-agency [335]. In addition to its implications in dynamic therapy, thinning of the precuneus can predict nonresponse to CBT in late-life depression [336].

The dACC has also been implicated in predicting response to CBT. Fu et al. (2008) showed that pretreatment activity within the dACC which was comparable to healthy controls predicted a successful therapeutic response [240], which the authors correlated with involvement of the area in tasks associated with potential loss of reward; thus, normal activity in the midst of a major depressive episode may be an indicator of resilience. While the dACC may be implicated in ruminative and excessive cognitive control, increased dACC-dlPFC connectivity (akin to increased rACC-dlPFC coupling) may be adaptive in the task of engaging in actively reworking modes of thinking in CBT, allowing for greater treatment benefits. Areas of the cingulate and PFC are also implicated in reward processing (which may serve as models for anhedonia), in particular relating to their connectivity with striatal regions. Two studies utilized behavioral activation treatment for depression (BATD), which is geared specifically towards having patients interact in a sustained manner with positively reinforcing stimuli, attempting to ameliorate reward processing dysfunction [337, 338]. In one, response to treatment was predicted by greater connectivity between the caudate-dACC and caudate-rACC, as well as by greater connectivity between right putamen and right OFC (refer to Table 4 for additional findings). Within the reward paradigm, the dACC is associated with estimating potential values of rewards and cognitively gauging whether to consider a determined action [337]. Thus, as alluded to earlier, selective hyperactivity within the dACC may facilitate cognitive reworking in MDD while maintaining active ability to process reward, predicting better outcomes. In addition to connectivity with basal ganglia, enhanced connectivity of the OFC (involved with assigning salience to stimuli, as well as with reward processing and feedback) with cortical regions was also shown to be a predictor of treatment [338].

This brief discussion highlights that certain preexisting network activation, while contributing to some dimensions of psychopathology, may also be adaptive in allowing for optimal engagement in therapy and maximizing the response to treatment.

### 4.4. Interpersonal Component as Modifier of Neural Circuitry

Though intuitive, one aspect of psychotherapy is the fundamentally dyadic nature of this treatment modality. In keeping with the argument put forth by this paper, early life adversity and lack of dyadic attunement can create a neural circuitry which effectively diverts one from accessing the intensity of the affective response and turns to more cognitive control mechanisms as a default form of coping, in effect evading a more integrated manner of thinking. This template can lead to subsequent psychopathology and more ingrained view of self and others, ruminating on themes of worthlessness, isolation, and mistrust with regard to the intention of others. Reactivity within subcortical areas tends to magnify these core beliefs, requiring modulation by higher cortical areas which may not be capable of effecting such change. Through the dyadic interaction of the psychotherapy setting, a new pattern of interpersonal relating can be fostered, one based on empathic attunement and allowing for exploration of highly sensitive themes in a safe setting. The establishment of this relationship can allow for a restructuring of these default modes of responding, creating greater fluidity between different PFC areas, in particular accessing areas involved with empathic responses (i.e., vmPFC), which also are instrumental in controlling subcortical areas which impose a bottom-up, affectively driven response to stimuli. Interestingly, one of the trending modalities for treatment of PTSD is the adjunct use of methylenedioxymethamphetamine (MDMA) in addition to psychotherapy; one of the principles behind such an addition is to facilitate the “empathogenic” abilities of the patient, allowing for greater interpersonal comfort and thus “open up” about traumatic experiences without overwhelming anxiety or vigilance about the therapist [339, 340]. A good degree of controversy surrounds generalizing this viewpoint across psychotherapeutic modalities, as discussed. One of the more consistent forms of therapy in which this medialization is appreciated is psychodynamically oriented psychotherapy. The focus on the interpersonal relationship in the room, honing in on transference dynamics may contribute to the more consistent findings of medial structure activation, given the “real-time” interpersonal component which is intrinsic to the technical approach.

As an interesting contrast, meditation practitioners (who engage in an inherently solo practice) show a deactivation of the amygdala through* increased* activity in the dlPFC and decreased activity in the mPFC, indicating that heightening attention, self-monitoring, and executive/cognitive control can decrease one's visceral response, in a manner disengaged from the interpersonal circuitry discussed before [341, 342]. A similar phenomenon has been observed in practitioners of mindfulness, which borrows some of the same principles of meditation [343].

## 5. Conclusions

This paper focused on multiple aspects of environmental input on epigenetic and neural circuitry components. It highlighted how early life adversity can lead to repression of key genes through methylation processes and to the template of concretized forms of thinking which can be internalized as default modes of negotiating the environment. The reparative effects of the latter will be contingent on the presence of a nurturing other who can allow for the patient to feel a level of attunement which may have been lacking earlier in life. The purported lack of containment by the primary caregiver as a child can have long-lasting effects on genetic, neuroendocrine, and circuitry levels and inform multiple arenas of psychopathology. However, there is notable plasticity within areas of the CNS, facilitated by gene malleability promoted by demethylation, something which can be observed with positive environmental input (e.g., exercise, positive social environments, and psychotherapy), bolstered by the plastic effects of psychopharmacologic agents (indeed, the epigenetic effects of medications are well recognized as a potential ancillary component to enhance psychotherapy) [344]. As imaging and genetic research grows in sophistication, predictors of treatment response (both pertaining to medications and psychotherapy) may allow for more tailored approaches to patient care [314, 345]. There are still some conflicting findings in the literature which have made some extrapolations somewhat tenuous, as resting-state functional imaging can differ from more dynamic task assessments; also, the expression of certain genes which control circulating stress hormones and those promoting neuroplasticity do not always show straightforward correlations with symptom response. This in part stems from inconsistent findings regarding the influence of cortisol on memory consolidation, as well as the complicated relationship between neurotransmitters and psychopathology (e.g., the relationship between BDNF and the serotonergic system), something which is strongly underlined by the concept of differential susceptibility. The continued input of the environment is paramount in determining how risk or resilience factors will translate as such, inviting our field to revisit such dichotomous concepts and appreciate the dynamic and ever-evolving nature of gene-environment interactions. A truism has become the importance of therapeutic rapport, and the progressive medialization of neural circuitry observed with numerous forms of psychotherapy found by researchers assessing responses in a number of psychiatric conditions illustrates that the empathic interpersonal element is a vital component to treatment which transcends technique, allowing for the safety and novelty of a containing setting to provide the patient with means to reach into the depths of their conflicts and not feel alone.

## Figures and Tables

**Table:** (a) Description of psychotherapy and neuroimaging studies: CBT for anxiety disorders^*∗*^.

Diagnosis	Study	Number of patients (intervention group)	Design	Treatment effects of psychotherapy
PD	Kircher et al. (2013)	42 (unmedicated)	fMRI 12 weeks of CBT	Normalization of hyperactivation in IFG. Greater connectivity between IFG and: amygdala, hippocampus, ACC, mPFC, and lPFC Decrease in activation in the amygdala, anterior insula, dACC, rACC, and vmPFC
	Lueken et al. (2013)	49 (unmedicated)	fMRI 12 sessions of CBT (twice-weekly for six weeks)	Normalized hyperactivation of pgACC and amygdala. Increase in hippocampal activation with stimulus contingency processing Enhanced ACC-amygdala coupling
	Straube et al. (2014) [144]	42 (unmedicated)	fMRI 12 sessions of manualized CBT Patient-guided and therapist-guided protocols were compared	In therapist-guided group, there was an increase in activation of the hippocampus, as well as a decreased connectivity between left IFG and left hippocampus

SoP	Furmark et al. (2002)	18 (unmedicated)	PET scan CBT for eight weeks (each session was 3 hours) Compared with citalopram-only group	CBT group: decrease in limbic, paralimbic, and PAG hyperactivation. Results maintained at one-year follow-up (multiple brain areas needed to be included to reach statistical significance). Citalopram group: decrease in thalamic hyperactivation (suggesting decreased sensory input into the amygdala); decrease in vPFC activation

SP	Paquette et al. (2003)	12 (unmedicated)	fMRI 4 sessions	Decrease in activation in dlPFC and parahippocampal gyrus
	Soravia et al. (2016)	8 (unmedicated)	fMRI Unspecified number of CBT sessions (though scanning done one month after treatment started)	Anticipation: decreased cerebral blood flow (CBF) in bilateral parahippocampal gyri, ventral anterior thalamus, Brodmann area 8, and the ACC Postprocessing phase: reduced CBF in the bilateral insula and motor cortex.
	Straube et al. (2006)	28 (unclear if medicated)	fMRI 2 sessions (4-5 hours each)	Decrease in activation in ACC and insula

GAD	Maslowsky et al. (2010)	7 (unmedicated)	fMRI 8 weeks of CBT Comparison group was patients on fluoxetine	Increase in activation in vlPFC to angry faces
	McClure et al. (2007)	12 (unmedicated) (3 with a diagnosis of social phobia)	fMRI 8 weeks of CBT	Decrease in amygdala activity (not statistically significant)
	Fonzo et al. (2014)	21 (unmedicated)	fMRI 10 sessions of CBT	Before treatment, patients showed blunted responses to positive faces in the amygdala, insula, and ACC; they also showed heightened amygdala-insula and amygdala-precuneus connectivity After treatment: decrease in activation to angry and fearful tasks in the sgACC and amygdala. Increase in activation to happy tasks in the anterior and posterior insula

SAD	Doehrmann et al. (2013)	39 (unmedicated)	fMRI 12 weeks of CBT	Activation changes in areas of lPFC, vPFC, and in the amygdala, none statistically significant
	Goldin et al. (2013)	75 (unmedicated)	fMRI 16 sessions of CBT	Cognitive reappraisal of negative self-beliefs was parameter assessed. Amygdala reactivity to negative self-beliefs remained consistent over time Increase in the dmPFC and dlPFC
	Goldin et al. (2014)	59 (unmedicated)	fMRI 16 weeks of CBT	Increase in the superior frontal gyrus, middle occipital lobe, and inferior parietal lobule activity when reacting to social praise Increases in right superior frontal gyrus and inferior parietal lobule, and decreases in left posterior superior temporal gyrus when reacting to social criticism
	Klumpp et al. (2013)	14 (2 on bupropion, the rest unmedicated)	fMRI 12 weeks of CBT	No significant correlation between symptom improvement and activation patterns in dmPFC or mPFC
	Yuan et al. (2016)	15 (4 on stable doses of SSRIs)	fMRI 8 weeks of group CBT	Attenuation of dACC-amygdala and dmPFC-amygdala connectivity

^*∗*^The following legend serves as a guide for all the tables in this paper.

d = dorsal; dl = dorsolateral; dm = dorsomedial; l = lateral; m = medial; pg = pregenual; r = rostral; sg = subgenual; v = ventral; vl = ventrolateral; vm = ventromedial.

ACC = anterior cingulate cortex; AG = agoraphobia; BATD = behavioral activation treatment for depression; BDI = Beck Depression Inventory; BG = basal ganglia; CAPS = clinician-administered PTSD scale; CBT = cognitive-behavioral therapy; DBT = dialectical behavioral therapy; EMDR = eye movement desensitization and reprocessing; fMRI = functional magnetic resonance imaging; GAD = generalized anxiety disorder; IFG = inferior frontal gyrus; MDD = major depressive disorder; OFC = orbitofrontal cortex; PAG = periaqueductal gray; PCC = posterior cingulate cortex; PD = panic disorder; PET = positron-emission tomography; PFC = prefrontal cortex; PTSD = posttraumatic stress disorder; rCBF = resting cerebral blood flow; SAD = social anxiety disorder; SERT = serotonin transporter; SoP = social phobia; SP = specific phobia; SPECT = single-photon emission computed tomography; SSRIs = selective serotonin reuptake inhibitors.

**Table:** (b) Description of psychotherapy and neuroimaging studies: CBT for major depressive disorder.

Diagnosis	Study	Number of patients (intervention group)	Design	Treatment effects of psychotherapy
MDD	Amsterdam et al. (2013)	20 (unmedicated)	SPECT 12 weeks of CBT (twice weekly for four weeks, then weekly)	Increase in standardized uptake ratio in the midbrain and bilaterally in the medial temporal lobes
	Fu et al. (2008)	16 (unmedicated)	fMRI 16 sessions of CBT	Less amygdala hyperactivity when exposed to sad faces. Normalization of amygdala-hippocampus activation pattern
	Goldapple et al. (2004)	17 (unmedicated; 14 completed protocol)	PET 15–20 sessions of CBT Comparison group was on paroxetine	Increased activity in dACC, hippocampus, and parahippocampal gyrus activity Decreased frontal cortical activity mainly in the dlPFC and OFC
	Kennedy et al. (2007)	12 (unmedicated)	PET 16 weeks of CBT (at least 8 weeks of treatment completed prior to rescanning; all but one patient completed 16 weeks) Comparison group treated with venlafaxine	Decrease in metabolism bilaterally in the PCC (opposite to venlafaxine group), OFC, and in the left dmPFC. Increase in metabolism in the right inferior occipital cortex, in the sgACC, and in the vmPFC
	Klein et al. (2014)	10 (1 medicated, stable dose of venlafaxine for months)	fMRI 12 weeks of CBASP^*∗*^ (mean number of sessions was 15.8; each session was 50 minutes)	
	Ritchey et al. (2011)	15 (unmedicated) Data from 11 patients was used	fMRI Weekly CBT sessions (average of 20.7 sessions and 30.3 weeks)	Increase in vmPFC activity
	Sankar et al. (2015)	16 (unmedicated)	fMRI 16 weeks of CBT	Decreased parahippocampal activity Increased activity in hippocampus, precentral gyrus, inferior parietal lobe, and precuneus
	Straub et al. (2015)	18 (unmedicated)	fMRI 5 sessions of group CBT	Decrease in bilateral amygdala, hippocampus, and sgACC activity
	Yoshimura et al. (2014)	23 (all on stable doses of antidepressants for at least 8 weeks)	fMRI 12 weekly sessions of group CBT	Activity during self-referential processing in vACC and mPFC was increased for positive stimuli and decreased for negative stimuli
	Yoshimura et al. (2017)	29 (all on stable doses of antidepressants for at least 8 weeks)	fMRI 12 weekly (90-minute) CBT sessions	Decrease in dysfunctional mPFC-ACC connectivity correlated with improvement on BDI score

^*∗*^CBASP = cognitive behavioral analysis system of psychotherapy.

**Table:** (c) Description of psychotherapy and neuroimaging studies: CBT for posttraumatic stress disorder.

Diagnosis	Study	Number of patients (intervention group)	Design	Treatment effects of psychotherapy
PTSD	Bryant et al. (2008)	14 (7 considered treatment responders, 3 of which were on psychotropics)	fMRI 8 weeks of CBT (including imagined and in vivo exposure)	After treatment, the CAPS score was positively correlated with amygdala and ACC activity
	Thomaes et al. (2012)	29 (some on stable doses of SSRIs or benzodiazepines)	fMRI 20 weekly sessions (group CBT)	Decrease in activation in anterior insula and dACC to emotional Stroop

**Table 2:** Description of psychotherapy and neuroimaging studies: miscellaneous psychotherapies.

Modality of psychotherapy	Diagnosis	Study	Number of patients (intervention group)	Design	Treatment effects of psychotherapy
Eye movement desensitization and reprocessing (EMDR)	PTSD	Bossini et al. (2011)	9 (unmedicated)	MRI 12 weeks (weekly 90-minute sessions)	Bilateral volume increase in the hippocampus
		Pagani et al. (2007)	15 (unmedicated)	SPECT 8 weeks (five 90-minute sessions)	Decreased tracer uptake in medial temporal cortex (uncus), hippocampus, occipitotemporal cortex, and visual cortex (BA 17)

Prolonged exposure (PE)	PTSD	Felmingham et al. (2007)	8 (2 on SSRIs; the rest unmedicated)	fMRI 8 weeks of treatment (cognitive restructuring with imaginal exposure)	Increase in rACC activity and a decrease in bilateral amygdala activity
		Helpman et al. (2016)	16 (unmedicated)	fMRI 10-week PE protocol	Decrease in rACC activation and increased activity in the mPFC

Brief eclectic psychotherapy (BEP)	PTSD	Lindauer et al. (2008)	20 (unmedicated)	SPECT 16 weekly sessions	Decrease in rCBF in the right uncus Increase in rCBF in the left superior temporal gyrus

Guided imagery	MDD	Huang et al. (2014)	23 (unmedicated)	fMRI 5 weeks of treatment	Increase in regional homogeneity in the vmPFC and ACC

Interpersonal psychotherapy (IPT)	MDD	Brody et al. (2001) [145]	39 (unmedicated)	PET 12 weeks of CBT (comparison group received paroxetine)	Increased activity in the dlPFC and dACC Decrease in activity in the vACC and anterior insula
		Brody et al. (2001) [146]	24 (unmedicated)	PET 12 weeks of IPT Comparison group received paroxetine	Reduced activity in lateral PFC, caudate, thalamus, left ACC Increased activity in left temporal lobe
		Martin et al. (2011)	10 (unmedicated)	SPECT Up to 16 hour-long weekly sessions (comparison group was treated with venlafaxine)	Increased blood flow in right BG and right PCC

Dialectical behavioral therapy (DBT)	BPD	Goodman et al. (2014)	11 (unmedicated)	fMRI 12 months of DBT	Decrease in amygdala activation to unpleasant images
		Paret et al. (2016)	8 (all patients on medications)	fMRI 12 weeks of DBT; amygdala neurofeedback (NF) incorporated for 4 sessions	Increased connectivity between vmPFC and amygdala (did not persist without NF component)

**Table 3:** Description of psychotherapy and neuroimaging studies: psychodynamic psychotherapy.

Diagnosis	Study	Number of patients (intervention group)	Design	Treatment effects of psychotherapy
PD	Beutel et al. (2010)	9	fMRI 4 weeks (inpatient setting)	Decreased limbic and increased vPFC and OFC activity Parameter was emotional linguistic go/no-go task

MDD	Buchheim et al. (2012)	16 (unmedicated)	fMRI 15 months	Decrease in amygdala, dmPFC, and sgACC hyperactivity
	Karlsson et al. (2010)	8 (unmedicated)	PET 16 weeks of therapy Comparison group received fluoxetine	Greater binding of 5-HT1A in the medial PFC and in the OFC
	Roffman et al. (2014)	9 (treatment completers; all on antidepressants)	PET 16 weeks of dynamic therapy using the Core Conflictual Relationship Theme manual	Decrease in metabolism of right insula
	Viinamäki et al. (1998)	1 (unmedicated) (Likely comorbid borderline personality disorder)	SPECT Weekly therapy for 1 year	SERT density normalization within the mPFC
	Wiswede et al. (2014)	18 (unmedicated)	fMRI 8 months	Decrease in BG and amygdala hyperactivity to operationalized psychodynamic diagnostics (OPD) sentences

BPD	Lai et al. (2007)	5 enrolled (2 completed) (unmedicated)	SPECT Weekly for 16 months	Increased perfusion in frontal cortical areas

**Table 4:** Description of psychotherapy and neuroimaging studies: predictors of therapeutic response.

Modality of psychotherapy	Diagnosis	Study (including design if not previously outlined)	Predictors of response
CBT	GAD	Ball et al. (2014) 48 patients (25 GAD, 23 PD), all unmedicated fMRI 10 weekly sessions of CBT	Greater activation in hippocampus during maintenance of emotional response to negative images Greater activation in anterior insula, superior temporal gyrus, supramarginal gyrus, and superior frontal gyrus during cognitive reappraisal
		McClure et al. (2007)	Pretreatment amygdala hyperactivity

CBT	SAD	Doehrmann et al. (2013)	Greater pretreatment activation to angry versus neutral faces in portions of the occipitotemporal cortices
		Klumpp et al. (2013)	Increased pretreatment activity to threat in the superior and middle temporal gyri and in the IFG. Hyperactivation to fearful faces in dmPFC, OFC, and dACC
		Klumpp et al. (2014) [147] fMRI 21 patients (unmedicated except for 2 on bupropion) 12 weekly 60-minute sessions	Increased pretreatment activity (in the presence of emotional faces) in the dACC and dmPFC Decreased pretreatment amygdala and mOFC activity during emotion processing
		Klumpp et al. (2014) [148] fMRI 21 patients (unmedicated except for 2 on bupropion) 12 weeks of CBT	Greater pretreatment bilateral amygdala-pgACC connectivity
		Mansson et al. (2015) fMRI/support vector machines (SVM) 26 patients (8 medicated) 13 weeks of Internet-delivered CBT	Pretreatment dACC activation and lower dACC-amygdala coupling during self-referential criticism task (92% balanced accuracy)^*∗*^ predicted sustained response one year later

CBT	PD/AG	Lueken et al. (2013)	Greater pretreatment coupling between ACC and amygdala
		Reinecke et al. (2014) fMRI 14 unmedicated patients with PD 4 sessions of CBT	Increased pretreatment activation in bilateral insulae and left dlPFC during threat processing Increased right hippocampus gray matter
		Straube et al. (2014) [149]	Nonresponse associated with heightened amygdala reactivity before and after treatment (informed by 5-HT1A rs6295 polymorphism G/G genotype)

CBT	MDD	Costafreda et al. (2009) 16 unmedicated patients fMRI 16 sessions of CBT	Response to treatment (gauged by response to sad faces) predicted by activity in the ACC, superior and middle frontal cortices, paracentral cortex, superior parietal cortex, precuneus, and cerebellum
		Crowther et al. (2015) 20 unmedicated patients Resting-state functional connectivity MRI Average of 12 sessions of BATD	Pretreatment connectivity of the insula with the right medial temporal gyrus and between the left intraparietal sulcus and OFC
		Fu et al. (2008)	Pretreatment activity in the dACC comparable to healthy controls predicted response
		Konarski et al. (2009) 7 unmedicated patients PET 16 sessions of CBT Comparison group was on venlafaxine	*Nonresponse* associated with pretreatment hypermetabolism the interface of the pgACC and sgACC
		Mackin et al. (2013) 22 patients (not receiving antidepressants) MRI 12 weeks of CBT	*Nonresponse *associated with thinner bilateral PCC and parahippocampal cortex, left paracentral, cuneus, and insular cortices, and right mOFC and lateral occipital cortex
		McGrath et al. (2014) 82 total patients (unmedicated prior to starting trial) PET 12–24 weeks of CBT and/or antidepressant (escitalopram) treatment	*Nonresponse* associated with pretreatment hyperactivity in the subcallosal cingulate and in the superior temporal sulcus
		Ritchey et al. (2011)	Increased pretreatment vmPFC and dlPFC activity predicted response. Parameter analyzed was response to pictures of different affective valence
		Siegle et al. (2006) 14 unmedicated patients fMRI 16 sessions of CBT (12 weeks)	Response to treatment was predicted by low sgACC reactivity and high amygdala reactivity to negative words
		Straub et al. (2015)	Pretreatment sgACC hyperactivity to positive (versus negative) stimuli predicted response
		Thompson et al. (2015) 60 patients (59 years and older; 44 completed study; 83% on at least one medication, though type not specified) fMRI 12 weeks of CBT	Decrease in activation in left inferior frontal triangle and right superior frontal gyrus Increase in activation of right middle frontal gyrus and left superior frontal gyrus
		Walsh et al. (2016) 33 unmedicated patients fMRI Up to 15 BATD sessions (average of 11.67 weekly sessions)	During anticipation phase, response was associated with: greater caudate-dACC and caudate-rACC connectivity; greater right putamen-right OFC connectivity; decreased connectivity between right mPFC and dACC; and decreased connectivity between left putamen and the subcallosal cortex
		Yoshimura et al. (2014)	Pretreatment decreased activity in vACC during self-referential information processing

CBT	PTSD	Bryant et al. (2008)	Pretreatment hyperactivity in amygdala, vACC, and dACC was associated with *nonresponse*
		Falconer et al. (2013) 13 patients (6 on stable doses of SSRIs) fMRI 8 weeks of CBT	Treatment response was predicted by higher pretreatment activation of frontostriatal networks (vlPFC, OFC, mPFC, and dorsal striatum) during inhibitory control tasks (this study employed Go/No-Go task)

Psychodynamic psychotherapy	MDD	Buchheim et al. (2012)	Increased pretreatment activity in the sgACC may predict response
		Roffman et al. (2014)	Pretreatment right precuneus metabolism (significantly higher in treatment completers; correlated also with psychological mindedness)

Guided imagery	MDD	Huang et al. (2014)	Increased pretreatment regional homogeneity within the dACC

EMDR	PTSD	Nardo et al. (2010) 20 patients (15 completed EMDR); unclear if patients were medicated or not (it was not an exclusion criterion) MRI Five 90-minute sessions	Decreased gray matter density in limbic and paralimbic cortices

^*∗*^Balanced accuracy is a mean of sensitivity and specificity.
