# Corrigendum to “Epigenetic and Neural Circuitry Landscape of Psychotherapeutic Interventions”

**DOI:** 10.1155/2017/6810473

**Published:** 2017-11-06

**Authors:** Christopher W. T. Miller

**Affiliations:** University of Maryland School of Medicine, 701 W. Pratt St., 4th Floor, Baltimore, MD 21201, USA

In the article titled “Epigenetic and Neural Circuitry Landscape of Psychotherapeutic Interventions” [1], there was an error in Table 1 and its legend. Under the Diagnosis column, the two rows of PD should be merged. Additionally, the study by Furmark et al. (2002) should be listed in the SoP row. The corrections have been made in place.

## References

[1] Miller C. W. (2017). Epigenetic and neural circuitry landscape of psychotherapeutic interventions. *Psychiatry Journal*.

